# A novel non prophage(-like) gene-intervening element within *gerE* that is reconstituted during sporulation in *Bacillus cereus* ATCC10987

**DOI:** 10.1038/s41598-017-11796-8

**Published:** 2017-09-12

**Authors:** Kimihiro Abe, Shin-ya Shimizu, Shuhei Tsuda, Tsutomu Sato

**Affiliations:** 1 Research Center of Micro-Nano Technology, Hosei University, Koganei, Tokyo, Japan; 2 Department of Frontier Bioscience, Hosei University, Koganei, Tokyo, Japan

## Abstract

Gene rearrangement is a widely-shared phenomenon in spore forming bacteria, in which prophage(-like) elements interrupting sporulation-specific genes are excised from the host genome to reconstitute the intact gene. Here, we report a novel class of gene-intervening elements, named *gin*, inserted in the 225 bp *gerE*-coding region of the *B. cereus* ATCC10987 genome, which generates a sporulation-specific rearrangement. *gin* has no phage-related genes and possesses three site-specific recombinase genes; *girA*, *girB*, and *girC*. We demonstrated that the *gerE* rearrangement occurs at the middle stage of sporulation, in which site-specific DNA recombination took place within the 9 bp consensus sequence flanking the disrupted *gerE* segments. Deletion analysis of *gin* uncovered that GirC and an additional factor, GirX, are responsible for *gerE* reconstitution. Involvement of GirC and GirX in DNA recombination was confirmed by an *in vitro* recombination assay. These results broaden the definition of the sporulation-specific gene rearrangement phenomenon: gene-intervening elements are not limited to phage DNA but may include non-viral genetic elements that carry a developmentally-regulated site-specific recombination system.

## Introduction

Bacterial endospore formation is an adaptive response to environmental changes such as nutrient exhaustion^1^. At the beginning of sporulation, asymmetric cell division produces two distinct cell types: the forespore and mother cell. The mother cell nurtures the forespore to maturity during spore morphogenesis, with a number of sporulation-related genes controlled at the transcriptional, translational, and posttranslational level^2, 3^. In such genetic regulatory systems, the gene rearrangement phenomenon represents different aspects, in terms of participation of exogenous genetic elements, of host gene expression control. In the process, an exogenous genetic element interrupting a sporulation-related gene is precisely excised from the host chromosome in a sporulation-specific manner, leading to the reconstitution of the intact and functional host gene^4–14^. To date, various gene-intervening elements have been found within sporulation-related genes in *Bacilli* ^11, 12, 14^ and *Clostridia* ^10, 13^, suggesting that gene rearrangement is a common and widely used phenomenon in spore forming bacteria. In particular, *sigK*-intervening elements (*skin*) are distributed among these bacterial strains^11^.

Gene-intervening elements that were previously reported to contain phage-related genes^9–11, 15^ are considered to be defective prophages that have lost the ability to produce infectious phage particles. This is supported by our recent report that both infectious and defective SPβ prophages in *Bacillus subtilis* and *B. amyloliquefaciens* can induce the rearrangement of *spsM*, whose gene product is involved in the production of spore surface polysaccharides^12, 14^. During gene rearrangement, the serine-type DNA recombinase encoded in the element recombines the disrupted gene segments through a site-specific recombination reaction^6–8, 12–14^. The reaction is most likely identical to phage excision, where the DNA recombinase recognizes and recombines the approximately 50 bp specific DNA motifs located upstream and downstream of the gene segments, which are called attachment sites (*attL* and *attR*), comprising the short direct and inverted repeat sequences^14, 16, 17^. The phage-encoded serine recombinases require an additional recombination directionality factor (RDF) for the excision^18^. Factors corresponding to the RDF encoded in gene-intervening elements have been reported in *B. subtilis* SPβ^12, 14^, and *Clostridium difficile skin* ^13^.

**Figure 1:**
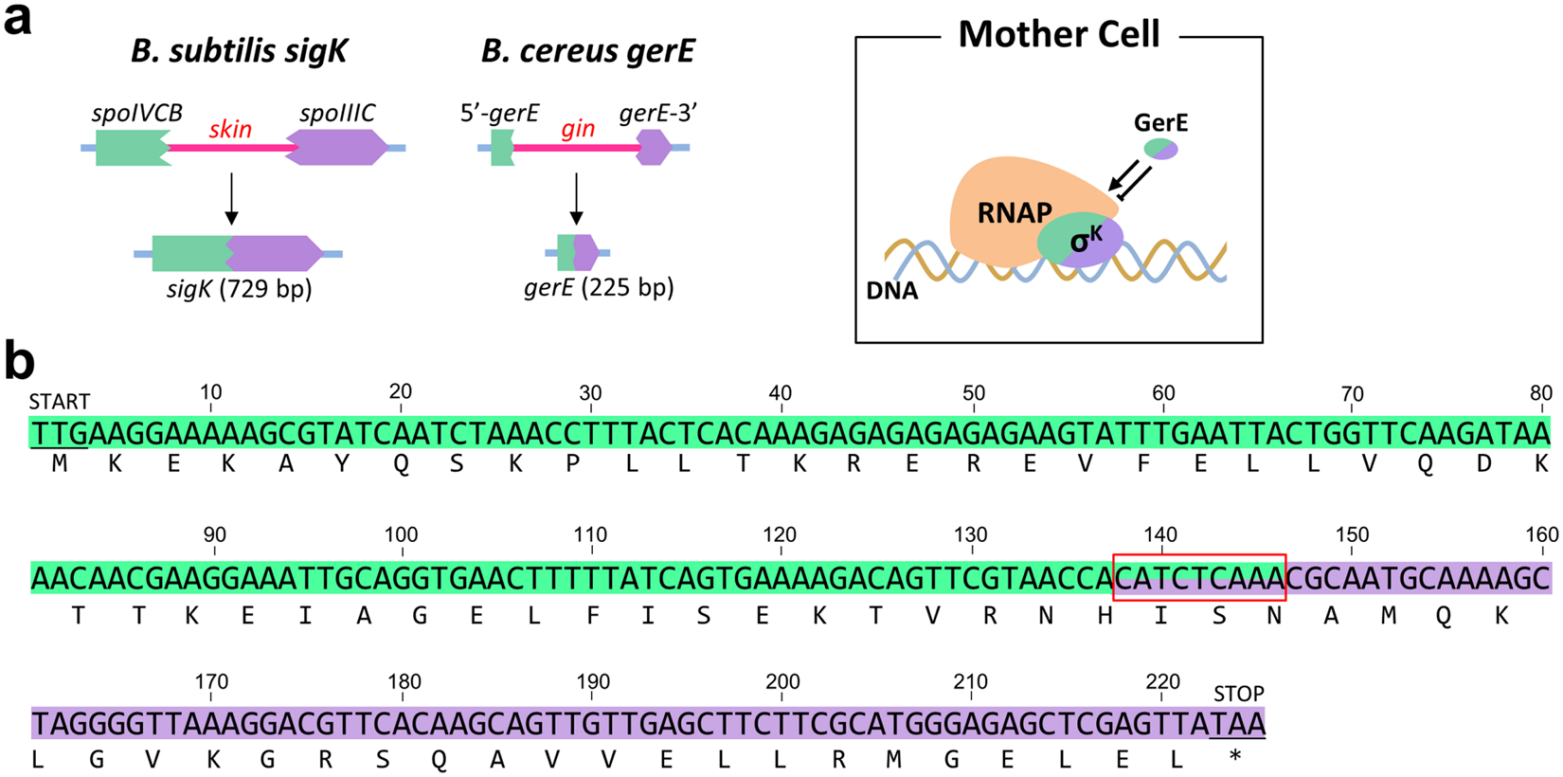
Gene rearrangement of *Bacillus cereus gerE*. (**a**) Schematic of gene rearrangements of *B. subtilis sigK* and *B. cereus gerE*. The composite *sigK* and *gerE* genes after the rearrangement encode a sporulation-specific sigma factor, σ^K^, and a transcriptional factor, GerE, respectively, which govern mother cell-specific gene expression at the late stage of sporulation. RNAP, RNA polymerase. (**b**) Whole nucleotide sequence of *gerE* from *Bacillus cereus* ATCC10987. Deduced amino acid sequence is shown under the nucleotide sequence. The *gin* element is inserted at position 138–146 nt (indicated by the red box). Nucleotides shaded green and purple correspond to the coding regions of 5′-*gerE* and *gerE*-3′, respectively. START, start codon; STOP, stop codon.

In this study, we investigated *gerE* gene rearrangement in *B. cereus* ATCC10987. GerE is a sporulation-specific transcriptional factor of 74 amino acids (aa) that modulates transcription of σ^K^-controlled genes in the mother cell compartment^19, 20^ (Fig. 1a), which is known to activate the transcription of the spore outer coat and germination receptor genes in *B. subtilis* ^21–23^. We found a 28.6 kb exogenous element splitting the 225 bp *gerE*-coding region, which contains no phage-related genes and possesses as many as three serine recombinase genes. These features are distinct from those of the prophage(-like) gene-intervening elements. In the previous study, we have defined that bacterial gene rearrangements are generated by intact or defective prophages^11, 12^. However, this novel type of gene-intervening element, named *gin*, provides us the opportunity to expand the definition. Moreover, such a small gene as *gerE* is targeted by the *gin* element implies the inevitability of interaction between the host sporulation system and the exogenous element in the evolutionary history. Therefore, analysis of the *gin* element seems important to deeply understand bacterial gene rearrangements and the evolution of sporulation. Here, we examined the rearrangement *in vivo* and *in vitro* and characterized this novel gene-intervening element.

## Results

### *Bacillus cereus gerE* is disrupted by an exogenous element

**Figure 2:**
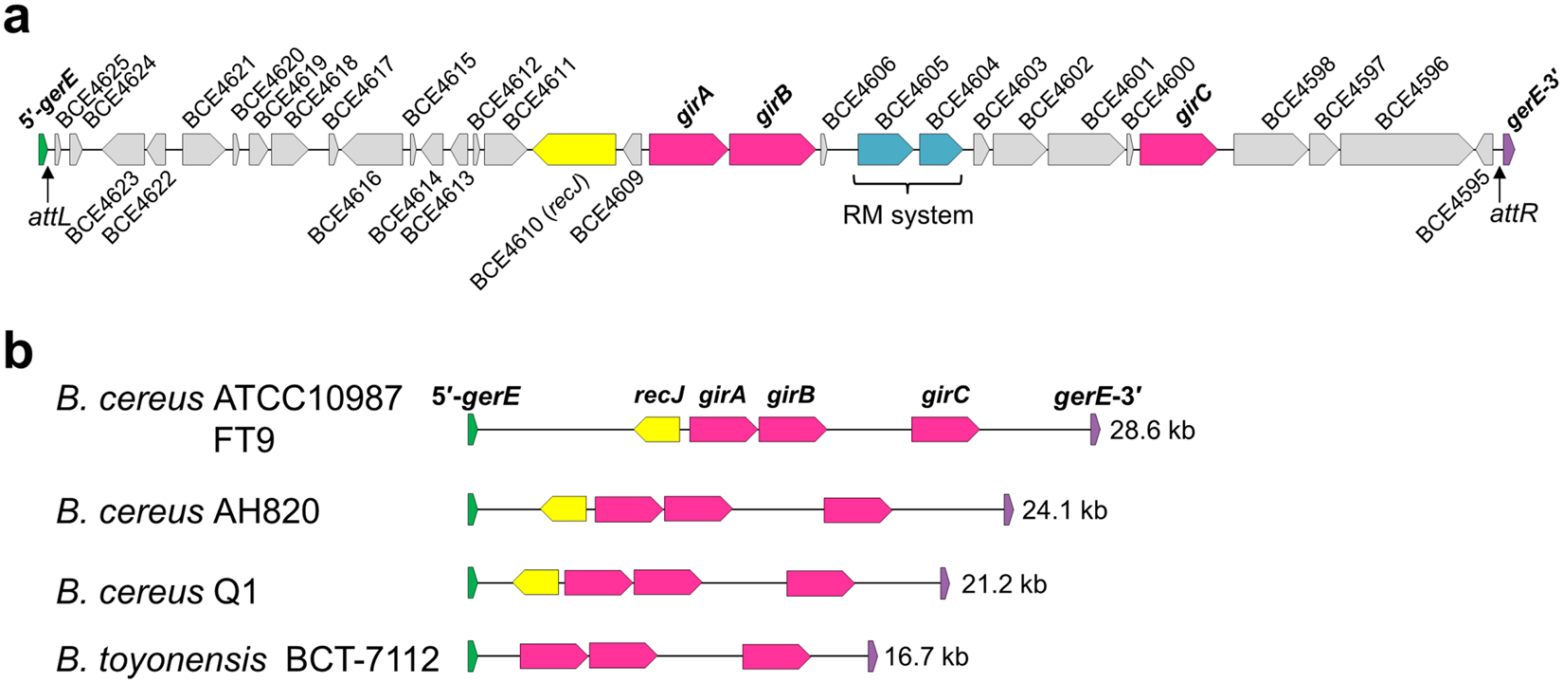
Genetic organization of the *gin* element and disrupted *gerE*. (**a**) The genetic map of the *gin* element is shown. Red, serine recombinase genes; blue, restriction-modification system genes; yellow, *recJ*. (**b**) Comparative genetic organization of *gin* elements in *Bacillus cereus* and *B. toyonensis*. The *gin* elements from *B. cereus* strains ATCC10987, FT9, AH820, and Q1 and from *B. toyonensis* BCT-7112 are shown. The size (kb) of the *gin* element is shown to the right.

*B. cereus* ATCC10987 *gerE* (*gerE*_*Bc*_) gene is composed of 225 nucleotides, which encodes a member of the LuxR-FixJ family transcriptional factors (Fig. 1a). The coding region is divided into two segments by an intervening element; the 5′ half (5′-*gerE*; formerly *BCE4626*; Fig. 1b, green) and 3′ half (*gerE*-3′; formerly *BCE4594;* Fig. 1b, purple) of *gerE*_*Bc*_ encode the 66 and 42 aa proteins corresponding to the N- and C-terminal regions of the entire GerE_Bc_, respectively. An overlapping sequence, 5′-CATCTCAAA-3′, is found at the end of 5′-*gerE* and at the beginning of *gerE*-3′ (Fig. 1b, box), which is expected to be the integration site of the intervening element. The intervening element, named *gin* (*gerE*-intervening), is an exogenous genomic island with a lower GC content compared with the *B. cereus* ATCC10987 genome^24^. The *gin* element consists of 31 genes (Fig. 2a), most of which have no significant homologies with previously characterized proteins except the restriction/modification system (*BCE4604* and *BCE4605*)^25^ and a *recJ* homologue (*BCE4610*). While the *B. cereus* ATCC10987 genome contains three prophage elements, the *gin* element is unlikely to be derived from prophages due to the lack of bacteriophage-related proteins (Fig. S1; Table S1). Another notable feature of the *gin* element is that it contains three serine recombinase genes unlike the prophage(-like) gene-intervening elements, which possess a single site-specific recombinase gene for gene rearrangement^11^. *girA* and *girB* (*gin*-encoded recombinase) form an operon, while *girC* is distant from the operon. The sequence homology for all three recombinases was low (approximately 22–26% identity; alignments of their amino acids sequences are shown in Supplementary Figure S2). The *gin* element is found in some *B. cereus* and *B. toyonensis* strains*;* the *gin* element in *B. cereus* FT9 is identical to that in ATCC10987 at the nucleotide sequence level (Fig. 2b). All of the *gin* elements identified possess all three recombinase genes even though their gene contents are different. In addition, *recJ* is also conserved in the *B. cereus* strains (ATCC10987, FT9, AH820, and Q1), suggesting that three recombinases and *recJ* may be required for maintenance or mobilization of the elements in *B. cereus*.

### Sporulation-specific *gerE* rearrangement in *B. cereus* ATCC10987

**Figure 3:**
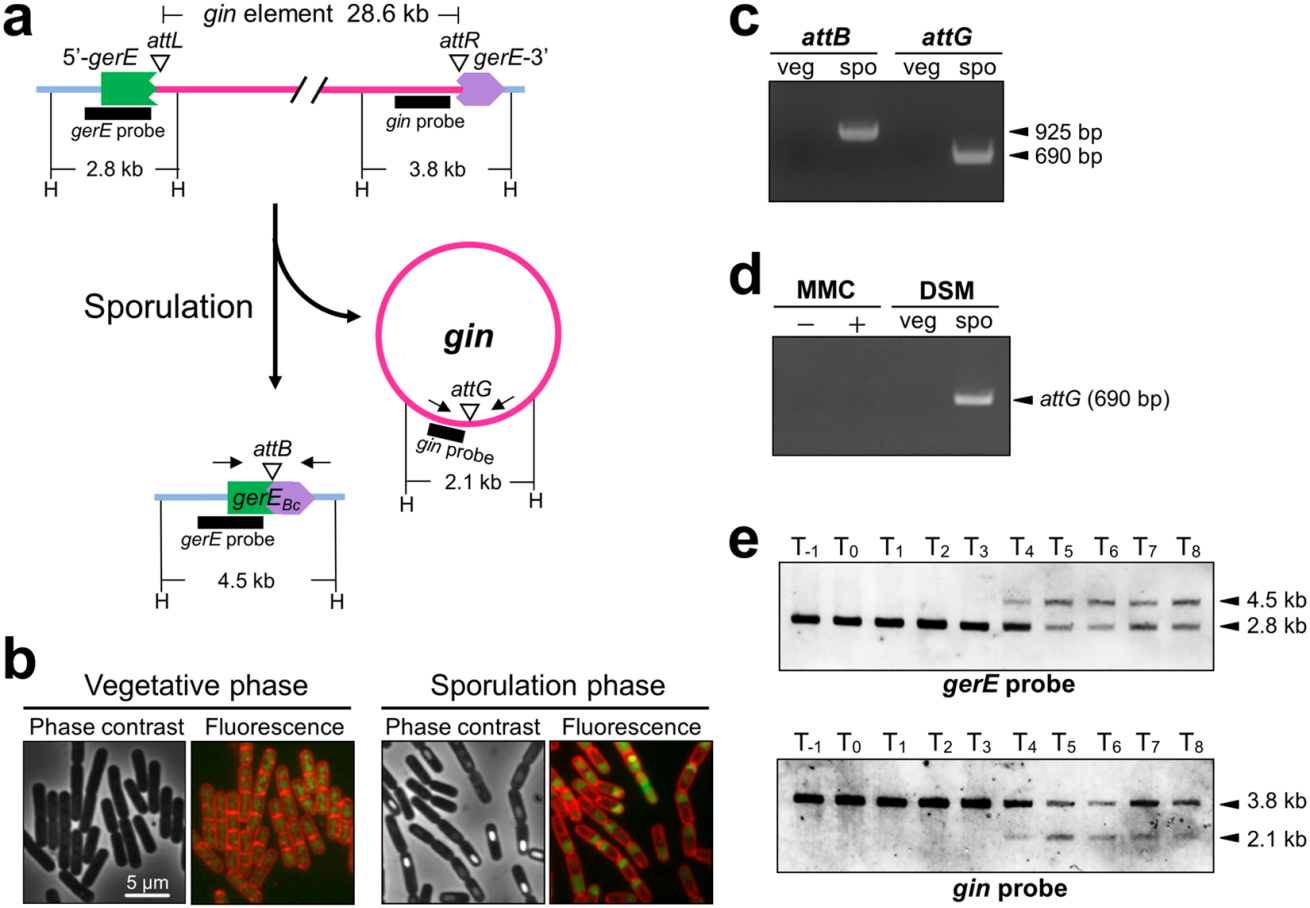
Verification of *gerE* rearrangement during sporulation in *B. cereus* ATCC10987. (**a**) Schematic showing the *gerE* locus before and after the rearrangement. H, *Hin*dIII restriction sites; triangles, DNA recombination sites; arrows, primers for PCR; thick lines, DNA probes for Southern blotting. (**b**) Cell morphologies of *B. cereus* vegetative and sporulating cells. *B. cereus* cells at T_-1_ (vegetative phase) and T_6_ (sporulation phase) were observed using phase contrast (left panel) and fluorescent microscopy (right panel). T_0_ was defined as the onset of the sporulation. The fluorescence images were generated by merging pictures of membrane staining with FM4-64 (red) and DNA staining with SYTO16 (green). Images from T_−1_ to T_12_ are shown in Supplementary Figure S3. (**c**) PCR detection of *gerE* rearrangement. Chromosomal DNA was isolated from *B. cereus* vegetative (T_−1_; veg) and sporulating (T_6_; spo) cells. The junction sites of the composite *gerE* (*attB*; 925 bp) and *gin* element (*attG*; 690 bp) were detected by PCR using the primers indicated in (**a**). (**d**) Insensitivity to mitomycin C (MMC). *B. cereus* cells were cultured at 37 °C in LB medium or sporulation medium (DSM). Vegetative cells (OD_600_ = 0.5) in the LB medium were treated with (+) or without (−) 0.5 μg/ml MMC for 90 min. The DSM culture was collected at T_−1_ (veg) and T_6_ (spo). The excised *gin* element was detected by PCR with the *gin*-specific primers. (**e**) Southern blotting. Chromosomal DNA from sporulating cells at various time points during sporulation was digested with *Hin*dIII, and subjected to Southern blotting using the *gerE*-specific (top panel) and the *gin*-specific (bottom panel) probes. The original gel and blot images of Fig. 3 are presented in Supplementary Figure S7.

Initially, we examined whether the *gerE* rearrangement occurred during sporulation in *B. cereus* ATCC10987. The 28.6 kb *gin* element was expected to be excised during sporulation as shown in Fig. 3a, given that the rearrangement created the intact *gerE* gene. *B. cereus* ATCC10987 cells were cultured at 37 °C in sporulation medium (DSM) to induce sporulation. The sporulation process in *B. cereus* ATCC10987 was very similar to that in *B. subtilis*. Engulfment began 3–4 hours after the onset of the sporulation (Fig. S3, T_3_, and T_4_), and bright forespores appeared in the sporangia at T_6_ and during the later stages. Consequently, free spores were released at T_12_. Chromosomal DNA was extracted from the vegetative (T_-1_) and sporulating (T_6_) cells (Fig. 3b; Fig. S3). The primers specific for 5′-*gerE* and *gerE*-3′ (Fig. 3a, arrows) were designed to amplify the DNA fragment containing the junction site of the composite *gerE* and the excised *gin* (Fig. 3a, *attB* and *attG*). The 925 bp PCR product was successfully generated only during the sporulation phase, but not during the vegetative phase (Fig. 3c, *attB*; Fig. S7a). Using the *gin*-specific primer set, the junction site of the excised *gin* element (*attG*; 690 bp) was also amplified only from chromosomal DNA in sporulating cells (Fig. 3c, *attG*, spo; Fig. S7a). However, the excision of *gin* was not induced when mitomycin C, a phage inducer, was added (Fig. 3d, MMC+; Fig. S7a), which is in contrast to the SPβ prophage-mediated *spsM* rearrangement in *B. subtilis* ^12^. These results suggested that the *B. cereus gerE* rearrangement is a sporulation-specific event. We then addressed the timing of the rearrangement during sporulation. Because the PCR-based assay was too sensitive to small differences in the sporulation state, we performed Southern blotting to determine the precise time point when the rearrangement commenced. Southern blotting using the *gerE* probe (Fig. 3a, thick line, *gerE* probe) detected a single 2.8 kb band, indicative of the 5′-*gerE*, starting during the vegetative stage (T_-1_) and up until three hours (T_3_) after the onset of the sporulation (Fig. 3e, top panel, T_-1_–T_3_; Fig. S7a). By contrast, at T_4_, a second 4.5 kb band began to appear and remained during later time points (T_4_–T_8_), indicating the rearrangement. At the same time, the excised *gin* element was detected (Fig. 3e, bottom panel, T_4_–T_8_, 2.1 kb band). These results indicate that the *gerE* rearrangement commenced at the middle stage of sporulation. Consistent with this, the *B. subtilis sigK* and *spsM* rearrangements have been reported to take place at the mid-phase of sporulation, after chromosome segregation and compartmentalization is complete^4, 5, 12^.

**Figure 4:**
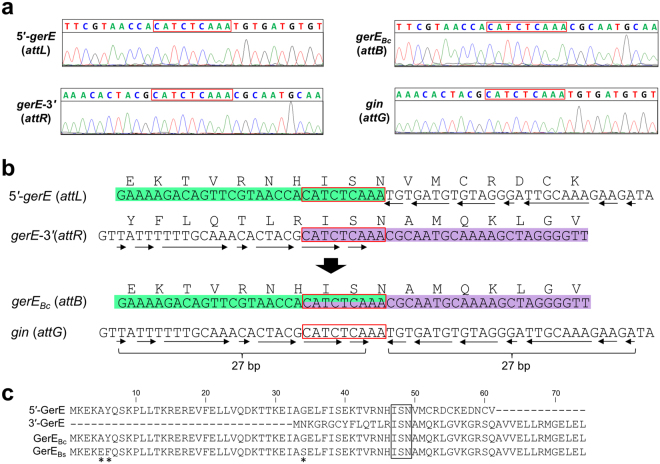
Nucleotide sequences of the DNA recombination sites. (**a**) DNA recombination sites before and after the *gerE* rearrangement were amplified by PCR and analyzed using a DNA-sequencer. The 9 bp consensus sequence between the DNA recombination sites is shown in the red box. (**b**) Nucleotide sequences at the DNA recombination sites before and after the rearrangement. The deduced amino-acids sequences of GerE are shown above the nucleotide sequence. The consensus sequence among the four *att* sites is indicated by the red box and the inverted repeat sequences are denoted by arrows. (**c**) Alignment of the amino acid sequences of the *B. cereus* and *B. subtilis* GerE proteins. The amino acid sequences encoded by the *B. cereus* truncated and composite *gerE* and *B. subtilis gerE* are shown as 5′-GerE, 3′-GerE, GerE_Bc_, and GerE_Bs_, respectively. The boxed amino acid sequences correspond to the region encoded by the 9 bp consensus sequence. Asterisks indicate the non conserved amino acids in *B. cereus* and *B. subtilis*.

To investigate whether the disrupted *gerE* segments were combined precisely, the four junction sites before and after to the DNA recombination were analyzed using a DNA sequencer (Fig. 4a). The sequencing data demonstrated that the nucleotide sequences of 5′-*gerE* and *gerE*-3′ were exchanged within the 9 bp consensus nucleotide sequence (5′-CATCTCAAA-3′; Fig. 4a, box), which encodes Ile-Ser-Asn at position 47–49 of the entire GerE_Bc_ (Fig. 4b). This suggests that DNA recombination takes place within the consensus sequence. The 27 bp imperfect inverted repeat sequence was found around the consensus sequence (Fig. 4b, arrows), which is likely to contain the recombinase binding sites. Alignments of GerE proteins showed that the *B. cereus* composite GerE (GerE_Bc_; 74 aa) was almost identical to *B. subtilis* GerE (GerE_Bs_) with only three substitutions at the 5^th^, 6^th^, and 34^th^ residues (Fig. 4c, asterisks), implying that the intact GerE_Bc_ was functional.

### DNA rearrangement produces a functional *gerE*

**Figure 5:**
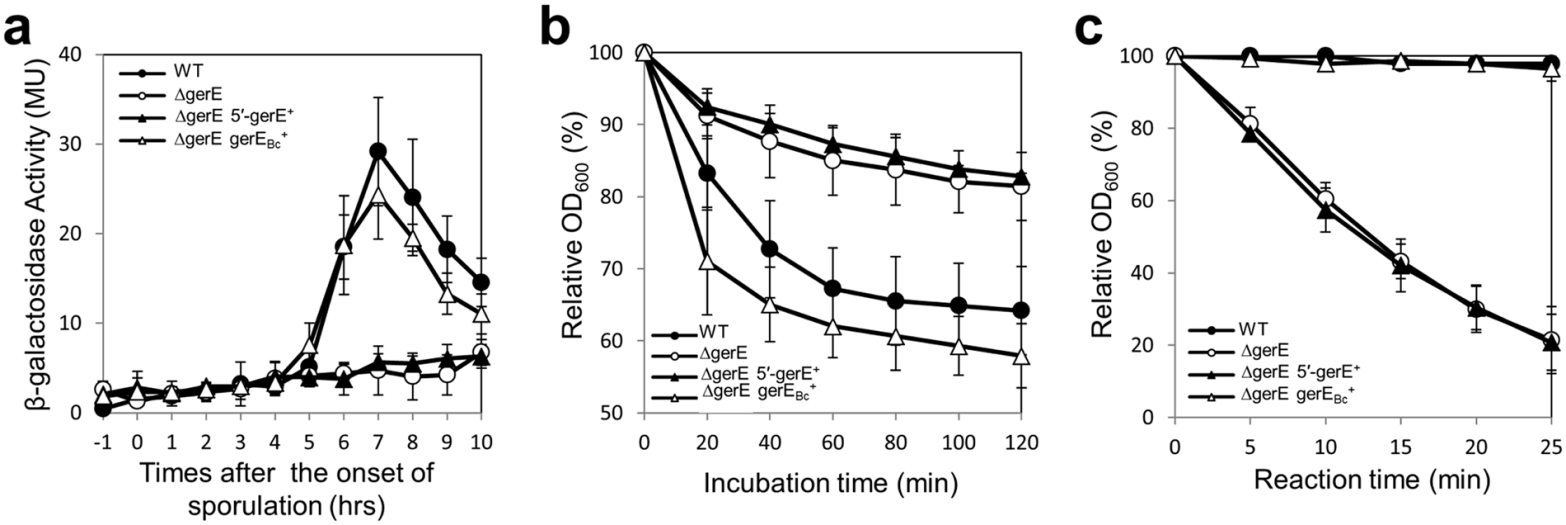
Gene complementation test for *gerE*. The *B. cereus* truncated (5′-*gerE*) and composite *gerE* (*gerE*_*Bc*_) were introduced into the *amyE* locus in the chromosome of the *B. subtilis gerE*-deletion mutant strain, GEd. (**a**) *lacZ* expression under control of the *cotG* promoter. A P_*cotG*_ *–lacZ* construct was introduced into *B. subtilis* 168 (wild type; filled circle), GEd (Δ*gerE*_*Bs*_; filled triangle), GEd-5 (Δ*gerE*_*Bs*_, 5′-*gerE* ^+^; open circle), and GEd-C (Δ*gerE*_*Bs*_, *gerE*_*Bc*_^+^; open triangle). The *B. subtilis* strains harboring the P_*cotG*_ *–lacZ* construct were induced to sporulate by cultivation in DSM at 37 °C. LacZ activity was measured at the indicated time points. (**b**) Germination rates. Spores from *B. subtilis* 168 (wild type; fill circle), GEd (filled triangle), GEd-5 (open circle), and GEd-C (open triangle) were treated at 70 °C for 30 min, and subsequently 1 mM L-alanine was added to induce germination. Relative OD_600_ values at each time point to the initial OD_600_ are plotted. Decrease in the OD_600_ reflects the initiation of germination. (**c**) Lysozyme sensitivity. Spores prepared from *B. subtilis* 168 (filled circle), GEd (filled triangle), GEd-5 (open circle), and GEd-C (open triangle) were treated with lysozyme at 37 °C. Relative OD_600_ values at each time point to the initial OD_600_ are plotted. A decrease in the OD_600_ value indicates the spore’s sensitivity to lysozyme. Error bars indicate the standard deviations based on three independent experiments.

In *B. subtilis*, GerE is required for the transcription of genes encoding spore outer-coat and germination receptor proteins, and depletion of the GerE activity results in the production of lysozyme-sensitive and germinant-insensitive spores^26^. To evaluate the activity of truncated and composite GerE *in vivo*, we conducted a *gerE* complementation test using the *B. subtilis gerE*-deletion mutant strain, GEd, and its derivatives harboring the truncated (5′-*gerE*; GEd-5) and composite *gerE* (*gerE*_*Bc*_; GEd-C). A P_*cotG*_–*lacZ* construct was introduced into the strains, generating 168 (wild type), GEd (Δ*gerE*_*Bs*_), GEd-5 (Δ*gerE*_*Bs*_, 5′-*gerE* ^+^), and GEd-C (Δ*gerE*_*Bs*_, *gerE*_*Bc*_^+^) strains to monitor the *cotG* promoter activity, which is positively regulated by GerE^23, 27^. During sporulation, *cotG* transcription began at T_5_ and reached its maximum at T_7_ in the wild-type strain (Fig. 5a, filled circle), while depletion of GerE_Bs_ failed to activate *cotG* transcription (Fig. 5a, open circle). This result was consistent with a previous report^27^. The composite *gerE*_*Bc*_ was able to compensate for the *gerE*_*Bs*_-deletion with a LacZ activity of 83.5% relative to that of the wild-type strain (Fig. 5a, open triangle), while 5′-GerE was not functional (Fig. 5a, filled triangle). A substitution in the 5^th^ residue of *B. subtilis* GerE has been reported to decrease the transcription rates from the GerE-dependent *cotC* promoter^22^. Therefore, the slight reduction in the *cotG* transcription level in GEd-C may have been caused by the differences in amino acid residues between GerE_Bs_ and GerE_Bc_ (Fig. 4c, asterisks).

Next, we examined the germination rates and lysozyme sensitivities of the spores produced from wild type, GEd, GEd-5, and GEd-C strains. Addition of a germinant, L-alanine (1 mM), decreased the OD_600_ values of the wild-type and GEd-C spores (Fig. 5b, filled circle and open triangle, respectively), which was a result of the reduced spore refractivity as germination was initiated. The wild type spore exhibited a 39% reduction in the OD value 120 min after the addition of L-alanine, whereas the GEd and GEd-5 spores showed only approximately 18% reductions (open circle and filled triangle, respectively), which indicated the insensitivity of the GEd and GEd-5 spores to the germinant. In addition, the GEd and GE-5 spores showed a significant loss of resistance to lysozyme (Fig. 5c, open circle and filled triangle, respectively). Taken together, these results indicate that the *B. cereus* composite GerE_Bc_ is functional, while the truncated 5′-GerE prior to DNA rearrangement has no activity as a transcriptional factor.

### GirC and GirX are required for *gerE* rearrangement

**Figure 6:**
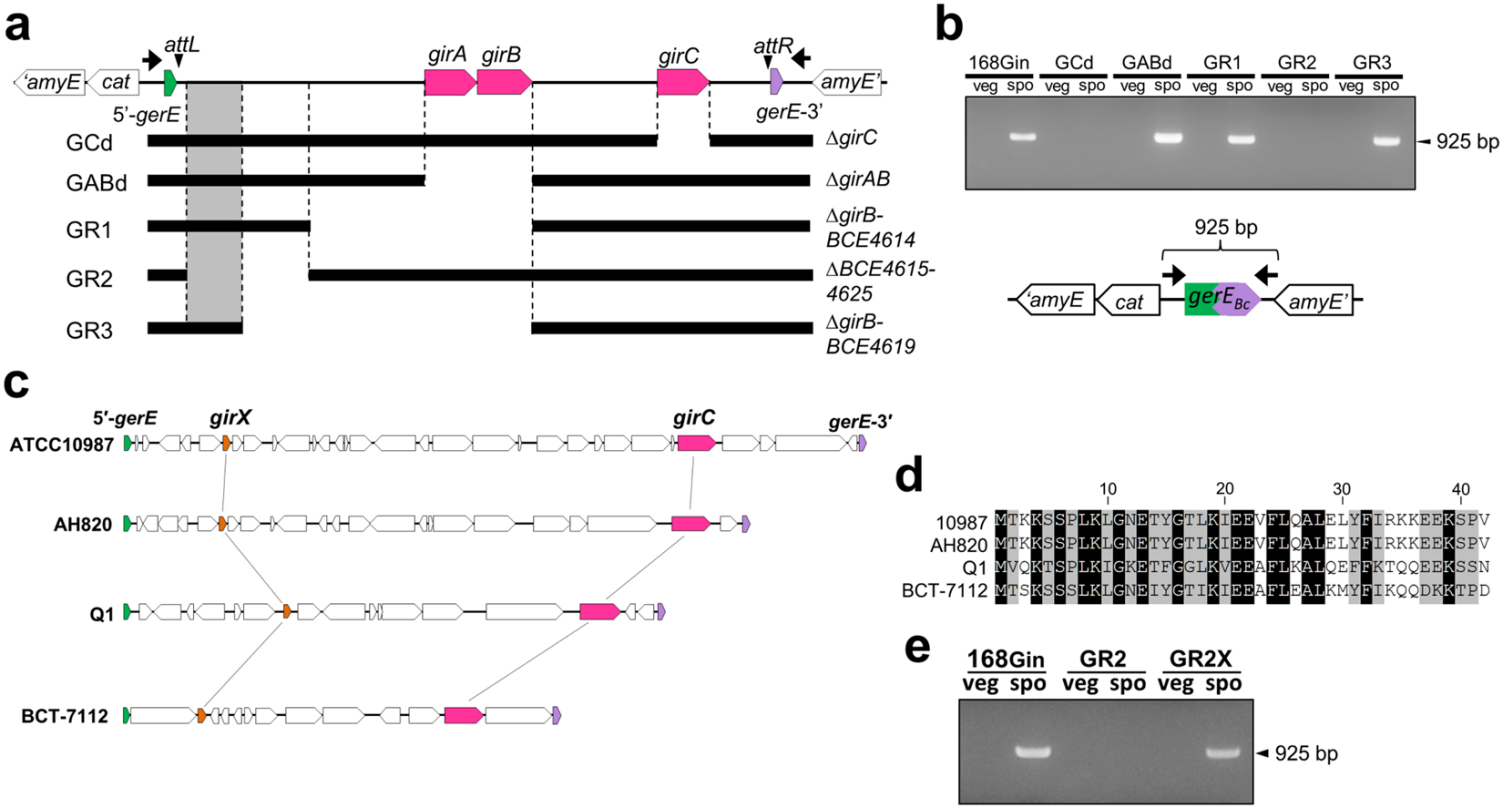
Identification of *gin*-encoded genes required for *gerE* rearrangement. (**a**) Schematic of the deletion series of the *gin* element. The deleted regions within the *gin* element in *B. subtilis* 168Gin are shown. Arrows indicate the PCR primers used to detect *gerE* rearrangement. The location of the putative RDF for GirC was inferred as the shaded area. (**b**) Detection of *gerE* rearrangement. PCR was performed to detect the composite *gerE* (925 bp) after rearrangement in 168Gin, GCd, GABd, and GR1–3 strains using chromosomal DNA from cells at the vegetative (T_−1_) and sporulation (T_6_) phases. (**c**) Conservation of *girX* in the *gin* elements in *B. cereus* and *B. toyonensis* strains. The *girC* and *girX* genes are colored red and orange, respectively. (**d**) Alignment of the amino acids sequences of GirX from *B. cereus* strains ATCC10987, AH820, and Q1, and from *B. toyonensis* BCT-7112. The amino acids conserved in three out of the four and in all four are shown in grey and black, respectively. (**e**) Complementation test for GirX. The *girX* gene was introduced into the GR2 strain (GR2X). PCR was performed to detect the composite *gerE* after the rearrangement under the same conditions as shown in (**b**). The original agarose gel images of Fig. 6 are presented in Supplementary Figure S7.

Previous studies described DNA recombination during sporulation-specific gene rearrangement events as catalyzed by serine recombinases encoded in the gene-intervening elements^5–8, 11–13^. Because *gin* carries three serine recombinase genes, we addressed which recombinase was responsible for the *gerE* rearrangement. Due to the difficulty in genetically manipulating *B. cereus* ATCC10987, the *B*. *cereus gerE* encompassing the entire *gin* element was introduced into the *amyE* locus of *B. subtilis* 168 (168Gin; Fig. 6a, top), which enabled the introduction of mutations into the *gin* element. In 168Gin, the *gin* element was capable of *gerE* reconstitution during sporulation (Fig. 6b, 168Gin, spo; Fig. S7b). Using 168Gin, we created the *girAB*- and *girC*-deletion strains (Fig. 6a, GABd and GCd, respectively). The *gerE* rearrangement was not observed in the *girC*-deletion strain (Fig. 6b, GCd, spo; Fig. S7b), while *girAB* was dispensable for the rearrangement (GABd, spo), indicating that only GirC was required for the rearrangement.

Overexpression of *girC* at the vegetative stage did not result in *gerE* rearrangement in a *girC*-inducible strain (Fig. S4, GC-i). This suggested that additional factor(s) for *gerE* rearrangement are controlled in a sporulation-specific manner. To localize the factor(s), GR1–3 strains, carrying partial deletions of *gin* elements, were constructed (Fig. 6a) and examined to determine their ability to induce the *gerE* rearrangement. As a result, GR1 and GR3 successfully generated the composite *gerE*_*Bc*_ (Fig. 6b, GR1 and GR3, spo; Fig. S7b), while GR2 showed no PCR product from the composite *gerE*_Bc_ (GR2, spo), indicating that the additional factor was encoded in a region from *BCE4620* to *BCE4625* (Fig. 6a, shaded area). Out of six genes in the *BCE4620*–*BCE4625* region, we found that *BCE4620* was conserved in the *gin* elements from *B. cereus* ATCC10987, AH820, Q1, and *B. toyonensis* BCT-7112 (Fig. 6c); hence, we designated *BCE4620* as *girX*. As shown in Fig. 6d, *girX* encodes a 42 aa protein with no conserved motif, but showed high similarity with the *gin*-encoded proteins from *B. cereus* AH820 (*BCAH4598*), Q1 (the corresponding ORF is not annotated in the public databases, but located at the intergenic region of *BCQ4304–BCQ4305*), and *B. toyonensis* BCT-7112 (located at the intergenic region of *Btoyo1736*–*Btoyo1737*). Introduction of *B. cereus* ATCC10987 *girX* into the *thrC* locus of GR2 recovered the rearrangement ability (Fig. 6e, GR2X; Fig. S7b). These results suggest that GirC and GirX are required for *gerE* rearrangement.

### Correlation of *girC* and *girX* expression with *gerE* rearrangement

**Figure 7:**
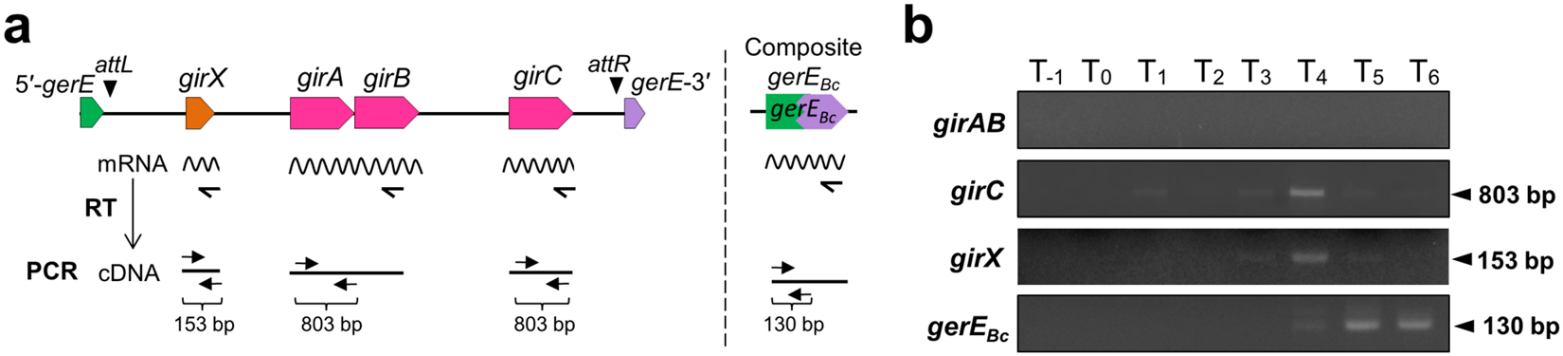
Transcription of *girC*, *girX*, and *gerE*_*Bc*_ in *B. cereus* during sporulation. (**a**) Diagram of the primer positions for the reverse transcription and PCR reactions. (**b**) Detection of the transcripts from the *girC*, *girX*, and the composite *gerE*_*Bc*_ gene. *B. cereus* cells were cultured at 37 °C in sporulation medium (DSM). Total RNA was extracted from the cells at various times during sporulation. cDNA was obtained by a reaction using reverse transcriptase with the *girC-*, *girX-*, and *gerE*_*Bc*_-specific primers, and amplified by 18 cycles of PCR with the appropriate primer sets shown in (**a**). The PCR products were separated by 2% agarose gel electrophoresis. The original agarose gel images are presented in Supplementary Figure S7.

The factors involved in *gerE* rearrangement should be expressed during the sporulation phase. The expression profiles of *girC*, *girX*, and *gerE* in *B. cereus* were determined using RT-PCR. Total RNA was isolated from *B. cereus* cells at various time points during sporulation, and *girC*, *girX*, and *gerE* cDNA was generated by reverse transcription using gene specific primers (Fig. 7a). PCR products from the *girC* and *girX* cDNA were detected at T_3_–T_5_ (Fig. 7b; Fig. S7c), and their signal intensities peaked at T_4_, which was consistent with the timing of *gerE* rearrangement (Fig. 3e). This indicates that the accumulation of sufficient amounts of GirC and GirX is required for the *gerE* rearrangement although the transcription begins at T_3_. The RT-PCR data suggests that the *gerE* rearrangement is a sporulation-specific reaction catalyzed by GirC and GirX. Transcription of the composite *gerE*_*Bc*_ occurred at T_5_ and T_6_, following the rearrangement (*gerE*_*Bc*_, T_5_ and T_6_). By contrast, *girAB* expression was not detected at any time between the vegetative state and sporulation phase (*girAB*, T_−1_–T_6_); hence, *girAB* expression may be induced under specific conditions other than sporulation.

### Establishment of the *in vitro gerE* rearrangement system

**Figure 8:**
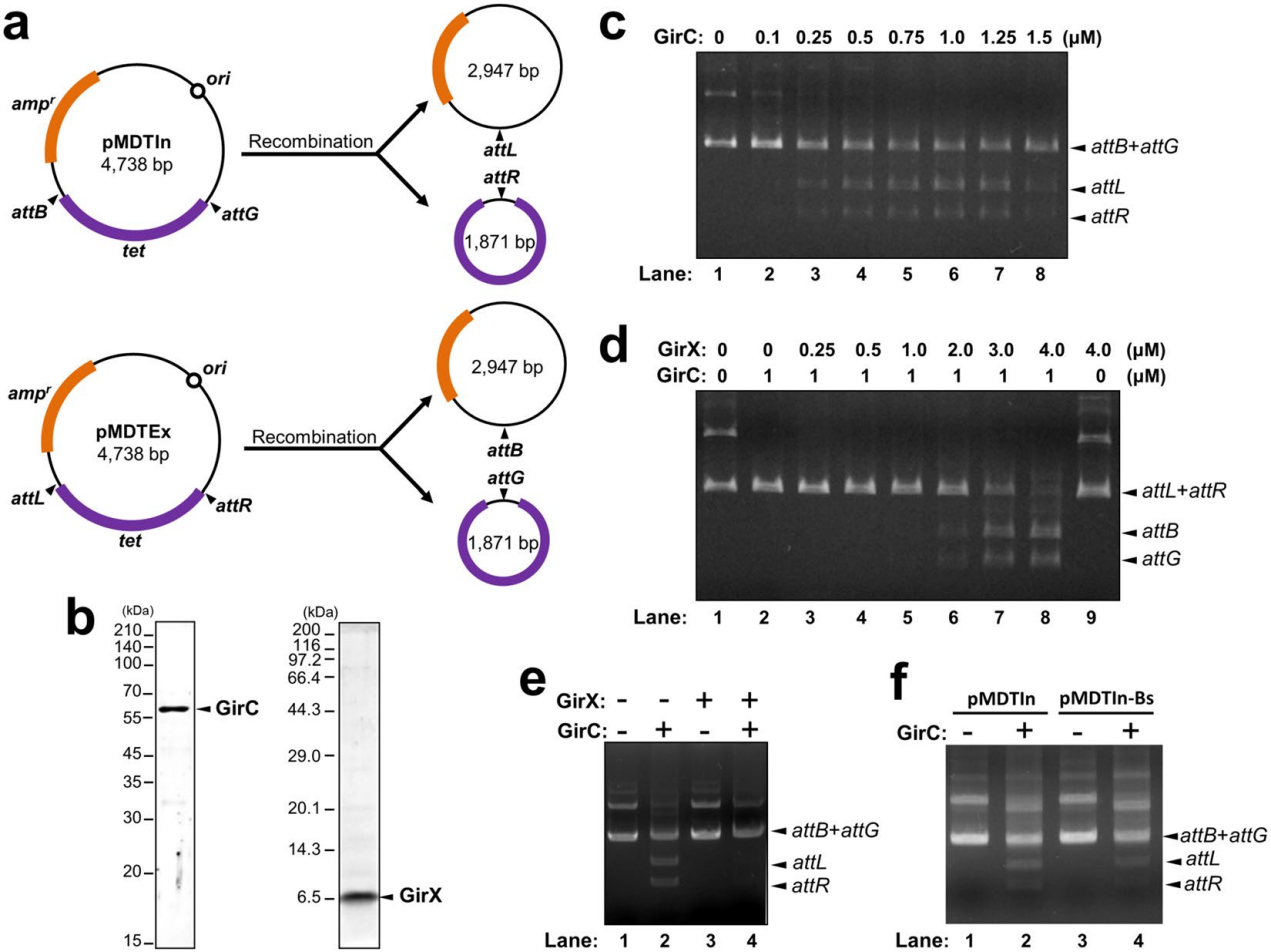
*In vitro* recombination assay. (**a**) Diagram of the *in vitro* recombination reactions. The *in vitro* recombination assays were performed using plasmid DNA carrying the *att* sites as the substrates for integration (pMDTIn) and excision (pMDTEx). (**b**) Purified GirC (1 μg) and GirX (1 μg) proteins fused with the his_6_-tag at their C-termini were loaded into SDS-PAGE (12%) and Tricine-SDS-PAGE (12%) gels, respectively. The original gel images are presented in Supplementary Figure S7. (**c**) Integration reaction. The DNA substrate, pMDTIn (200 ng; *attB* + *attG* [supercoiled]), was reacted with GirC-His_6_ (0–1.5 μM) at 37 °C for 1 hr. The recombination products, *attL* and *attR*, were analyzed by agarose gel electrophoresis. (**d**) Excision reaction. The DNA substrate, pMDTEx (200 ng; *attL* + *attR*), was reacted with GirC (0 or 1 μM) and GirX (0–4 μM) at 37 °C for 1 hr. The recombination products were analyzed by agarose gel electrophoresis. (**e**) Inhibition of integration by GirX. The integrative substrate, pMDTIn was incubated at 37 °C for 1 hr in the absence (−) or the presence of GirC (+; 1 μM) and GirX (+; 4 μM). The recombination products were analyzed by agarose gel electrophoresis. **(f)** *In vitro* recombination between *B. subtilis gerE* (*attB*_*Bs*_) and *attG*. The *attB* site of pMDTIn was replaced with *attB*_*Bs*_ to generate pMDTIn-Bs. pMDTIn-Bs was reacted with 1 μM GirC at 37 °C for 1 hr. Arrowheads indicate the recombination products, *attL* and *attR*. The original agarose gel images of Fig. 8 are presented in Supplementary Figure S7.

To verify the GirC/GirX-mediated *gerE* rearrangement, an *in vitro* recombination system was established. As shown in Fig. 8a, we constructed two plasmids harboring either the *attB*–*tet*–*attG* (pMDTIn) or *attL*–*tet*–*attR* (pMDTEx) sites to use as DNA substrates. The site-specific DNA recombination reaction between *attB* and *attG* in pMDTIn (or *attL* and *attR* in pMDTEx) was expected to produce two circular DNA molecules of 1,871 bp containing *attR* (or *attG*) and 2,947 bp containing *attL* (or *attB*). Recombinant GirC and GirX fused with a his_6_-tag at their C-termini were expressed in *E. coli* and purified by affinity chromatography. The purified GirC and GirX were detected as single 58 kDa and 6.5 kDa bands, respectively (Fig. 8b; Fig. S7d). pMDTIn (200 ng) was reacted with various amounts of GirC (0–1.5 μM) at 37 °C for 1 hr, and separated by agarose gel electrophoresis. Signals of the integrative recombination products, the circular DNA containing *attL* and *attR*, were detected in a GirC-dose dependent manner, and peaked at the GirC concentration of 1 μM (Fig. 8c; Fig. S7d). Excessive amounts of GirC decreased the yields of the recombination products (Fig. 8c, 1.25 and 1.5 μM GirC), which was consistent with previous reports on phage-encoded serine recombinases^14, 28^. In the excision reaction, pMDTEx (200 ng) was reacted with various amounts of GirX (0–4 μM) in the presence of GirC (1 μM) at 37 °C for 1 hr. The recombination products were detected when both GirC and GirX were added to the reactions (Fig. 8d, 2–4 μM GirX; Fig. S7d). The addition of 4 μM GirX generated the recombination product most efficiently when mixed with 1 μM GirC (lane 8). By contrast, neither of them showed excision activity when alone (lanes 2 and 9). This supports the results shown in Fig. 6. Unlike the excision reaction, the integration reaction using pMDTIn was only catalyzed by GirC (Fig. 8e, lane 2; Fig. S7d); the addition of GirX blocked the integrative recombination (lane 4). These results are consistent with phage-medicated site-specific recombination, whereby phage integrases catalyze the recombination between phage and host DNA and the recombination directionality is controlled by an additional recombination directionality factor (RDF)^16, 18^. We therefore concluded that GirC is the integrase that catalyzes the recombination between the *gin* element and the host *gerE* locus, and that GirX is the RDF for GirC. Furthermore, we found that GirC catalyzed the integration reaction between *attG* and *B. subtilis gerE* (*attB*_*Bs*_) with approximately 40% efficiency compared with the recombination with *B. cereus gerE* (Fig. 8f, lane 4; Fig. S7d). Surprisingly, the integration site was identical to that of *gerE*_*Bc*_, although differences in nucleotides were found around the recombination site (Fig. S5, red font). The *in vitro* study raised the possibility that the *gin* element is potentially mobile between *B. cereus* and *B. subtilis*.

## Discussion

In the present study, we demonstrated *gin*-mediated *gerE* rearrangement in *B. cereus*. Sporulation-specific expression of *girC* and *girX* (Fig. 7) induced site-specific DNA recombination (Figs 3 and 4) to produce the functional *gerE* (Fig. 5). Our results indicate that the gene rearrangement system encoded in the exogenous element is successfully incorporated into the gene expression program during sporulation in *B. cereus*.

We found that the *att* sites for GirC contain a 27 bp imperfect inverted repeat and the 9 bp consensus sequence (Fig. 4b, arrows and box). Generally, *attP* sites for phage-encoded serine recombinases comprise a symmetric sequence of the inverted repeat motif with respect to the central dinucleotides^16, 17^. The inverted repeat motif is the recognition site of the recombinase and DNA cleavage occurs at the central dinucleotides during DNA strand exchange. In the case of *gin*, the DNA cleavage site is predicted to be the 3′-end diadenine nucleotides within the 9 bp consensus sequence (Fig. 4b, *attG*, 5′-CATCTCAAA-3′; underlined) at the middle of the *attG* site inverted sequence.

In *gerE* rearrangement, an additional factor, GirX, was required in combination with GirC (Fig. 6), which controlled the directionality of the GirC-mediated recombination reaction (Fig. 8). These data demonstrated that *gerE* rearrangement occurs in a similar manner to prophage-mediated gene rearrangements, such as *B. subtilis* SPβ prophage^12^ and *C. difficile* prophage-like *skin*_*Cd*_^13^, in which serine recombinases play the central role in the reaction. However, there are differences in regulation; the serine recombinases are expressed constitutively in *B. subtilis* SPβ^12^ and *C. difficile skin*_*Cd*_^13^, while being expressed specifically at the sporulation phase for the *B. subtilis skin* ^8^, *B. weihenstephanensis vfbin* ^11^, and *B. cereus gin* elements. In the cases of *B. subtilis* SPβ and *C. difficile skin*_*Cd*_, their RDFs are under the control of sporulation-specific sigma factors. The *B. cereus gin* element proves that non-viral mobile elements, not only prophage-like elements, can be gene-intervening elements if they harbor the site-specific recombination system and are integrated into sporulation genes. As an interesting example, a prophage can be found integrated into the *gerE* locus in *B. glycinifermentas* BGLY (GenBank accession number, LT603683). If the rearrangement is verified in the strain, it will strongly support the idea that both mobile elements and prophages have the potential to induce gene rearrangements. A question of why those mobile elements targeted the *gerE*-coding region, which consists of only 225 nucleotides, is an important issue of interest. In the previous study on *spsM* rearrangement, we argued that *spsM* was targeted by SPβ phage because a unique symmetric sequence within the *spsM*-coding region was the preferred integration site of the phage integrase, SprA^14^. Unlike *spsM*, no symmetric sequence was found at the *attB* site (Fig. 4b); however, the *attG* site was probably selected as the recombination site because of the inverted repeat encompassing the 9 nucleotide sequence shared with the *gerE*-coding region. Functionally, GerE activates the late mother cell-specific genes, including *spsM*, while it represses σ^K^ expression^20, 23^. Therefore, *gerE* disruption by mobile elements would be beneficial for the host to block expression leakage at earlier stages during sporulation, which could disturb the gene expression program. Such physiological benefits for the host might have been given priority over the nucleotide preference of mobile element-encoded recombinases, followed by adaptation of the recombinases to the precise site recognition in the evolutionary history.

The *gin* element in *B. cereus* ATCC10987 is identical at the nucleotide level to the element in the thermotolerant *B. cereus* strain, FT9^29, 30^. This implies that the *gin* element was transferred across the strains very recently; however, the transfer mechanism is still unknown. The non-viral element, *gin*, may have spread among the strains by horizontal gene transfer, such as conjugation and natural competence. In *B. cereus* ATCC10987, the *gin* element itself has no structural genes for conjugation, while the host plasmid, pBc10987, carries a series of genes for conjugative transfer-like proteins^29^. The *gin* element, therefore, might be horizontally transferred in association with the plasmid-borne conjugation system. From the results of the *in vitro* recombination assay (Fig. 8), it is plausible that the *gin* element is integrated following transfer into the new host genome via the GirC-catalyzed DNA recombination. As shown in Fig. 7b, weak expression of *girC* was observed at T_1_, while the *girX* expression was not. This suggests that *girC* may be controlled by multiple regulation mechanisms, which would be necessary for the *gin* element to integrate into new host genome. Control of the transfer is also a significant issue. In *B. subtilis*, an integrative and conjugative element, ICE*Bs*1, employs ImmR and ImmA to control excision and transfer^31, 32^. ImmR is a transcriptional factor that represses the transfer, while ImmA is a metalloproteinase that specifically degrades ImmR. In response to DNA damage and cell-to-cell signaling, ImmA is activated to lyse ImmR, thereby causing derepression of ICE*Bs*1 excision, and consequently, transfer. Homologues of ImmR and ImmA are found in many mobile elements^31^. In the *gin* element, *BCE4623* and *BCE4624* encode a putative transcriptional factor and a metalloproteinase, respectively, and therefore are considered as candidates for the regulators of excision, or may be remnants of the conjugation system derived from the ancestor of the *gin* element.

Because *girAB* was not expressed under our conditions and was dispensable for the *gerE* rearrangement, the role of *girAB* remains to be clarified. The GirA and GirB from *B. toyonensis* show 83–86% identity with the serine recombinases from the *B. thuringiensis* MC28 plasmid, pMC319^33^, while GirC has 52% identity with the integrase encoded by the *B. glycinifermentas* prophage inserted in the *gerE* locus (Fig. S6). The *gin* element is likely to be a composite of mobile elements derived from the pMC319 plasmid and the prophage. In addition, GirC and GirX are highly conserved in *B. cereus* and *B. toyonensis* strains (81–85% identity), whereas GirA and GirB from *B. cereus* strains have lower homologies with those from *B. toyonensis* (approximately 30% identity), indicating that *B. cereus girAB* was derived from mobile elements other than pMC319. Well-known examples of composite mobile elements include Staphylococcal chromosomal cassettes (SCCs)^34, 35^, which are clinically significant genetic elements because they convey methicillin resistance genes (*mec* complex) among Staphylococcal strains. The serine recombinase genes encoded in SCCs are called chromosomal cassette recombinase (*ccr*). The SCCs are classified based on variations in the *ccr* and *mec* complex; however, composites of different SCCs are also found^34, 36^. *ccrA* and *ccrB* are always found together as a two-gene operon in SCCs. Their gene products can catalyze both integration and excision reactions without the RDF, and can also recombine canonical pairs of *att* sites (e.g., *attB* × *attS*_*CC*_ and *attL* × *attR*) and non-canonical pairs (e.g., *attS*_*CC*_ × *attS*_*CC*_ and *attR* × *attR*), which is thought to allow the fusion of multiple SCCs^37^. From this viewpoint, *girAB* may have contributed to composite *gin* elements. Alternatively, because *girAB* are encoded in pMC319, they might be a multimer resolution system for circular DNA. Further functional analysis of *girAB* will be required to understand the mechanism of horizontal gene transfer and co-evolution of the *gin* element and the host.

## Methods

### Bacterial strains and plasmids

The bacterial strains and plasmids used in this study are listed in Table S2. Primers used in this study are shown in Table S3. *Bacillus cereus* and *B. subtilis* strains were routinely incubated at 37 °C in Luria-Bertani (LB) medium with shaking. Transformation of *B. subtilis* was performed through natural competence. Detailed description of *B. subtilis* strain construction is presented in Supplementary Methods. Plasmids were constructed using the *E. coli* strain DH5α. For culture of *E. coli* strains harboring plasmids, 50 or 100 μg/ml ampicillin was added to the LB medium.

### Sporulation of *B. cereus* and *B. subtilis*

Overnight cultures of *B. cereus* and *B. subtilis* strains grown at 37 °C in liquid LB medium were diluted 1:100 into fresh liquid Difco sporulation medium (DSM; Becton, Dickinson and Company, MD, USA), and incubated at 37 °C with shaking to induce sporulation.

### Fluorescence microscopy

*B. cereus* cells were cultured in DSM containing FM4-64 (2.5 μg/ml) and SYTO16 (66 nM) to stain the cell membrane and chromosomal DNA, respectively. Microscopic observation was performed using an Olympus BX50 microscope with a 100× Uplan Apo objective lens. Images were captured and processed, using Metamorph software version 7.6.5 (Metamorph Inc., TN, USA).

### Detection of the *gerE* rearrangement

Chromosomal DNA was extracted from the *B. cereus* vegetative and sporulating cells, according to the method described previously^11^. The junction sequences at the *attB* (composite *gerE*) and *attG* (the excised *gin* element) sites were amplified from 100 ng of the chromosomal DNA with the primer sets PA246/PA249 and PA247/PA248, respectively. PCR products were separated by agarose gel electrophoresis, and stained using EZ-Vision DNA Dye (AMRESCO, OH, USA). Gel images were cropped, using Paintgraphic2 (SOURCENEXT, Tokyo, Japan). DNA sequencing was performed, using an ABI 3500 DNA analyzer (Thermo Fisher Scientific, WI, USA) with the PA246 (for the 5′- and composite *gerE*) or PA248 (for 3′-*gerE* and the excised *gin* element) primers and the BigDye^®^ Terminator v3.1 Cycle Sequencing Kit (Thermo Fisher Scientific).

### Southern blotting

To generate the digoxigenin (DIG)-labeled probes, DNA fragments corresponding to parts of the *gerE* and *gin* element adjacent of the junction sites were amplified from the *B. cereus* chromosomal DNA with the primer sets PA246/PA306 and PA309/PA310, respectively. PCR products were gel-purified and labeled by the incorporation of DIG-11-dUDP using the DIG High Prime kit (Roche, Mannheim, Germany). Five micrograms of chromosomal DNA from *B. cereus* was digested by *Hin*dIII overnight, separated by 1.2% agarose gel electrophoresis, and transferred onto a Hybond-N^+^ membrane (GE Healthcare, NJ, USA) by the capillary method using 10 × SSC. Hybridization and detection were performed using the DIG-labeled probes, anti-DIG antibody conjugated to alkaline phosphatase (Roche), and a nitro-blue tetrazolium/5-bromo-4-chloro-3-indolyl-phosphate solution (NBT/BCIP; Roche). Blot images were cropped and converted to gray scale, using Paintgraphic2 (SOURCENEXT).

### β-galactosidase assay

The 168-Z, GEd-Z, GEd-5Z and GEd-CZ strains were sporulated at 37 °C in liquid DSM. The cultures were collected at various time points once the exponential phase of growth had ended. β-galactosidase activity was measured using the method described previously^38^.

### Lysozyme sensitivity assay

Spores from *B. subtilis* 168, GEd, GEd-5, and GEd-C were purified as described previously^12^, resuspended in distilled and deionized water (DDW), and adjusted to 0.5 optical density at 600 nm (OD_600_). The spore resuspensions were incubated at 37 °C in the presence of lysozyme at a final concentration of 250 μg/ml. Lysozyme sensitivity was determined by a decrease in the OD_600_.

### Germination assay

The spore resuspensions (OD_600_ = 0.5) were heated at 70 °C for 30 min, 1 mM L-alanine was then the added and further incubated at 37 °C. Germination rates were determined by measuring the decrease in the OD_600_.

### RT-PCR

Total RNA was extracted from *B. cereus* sporulating cells using glass beads as described previously^39^. For synthesis of cDNA, 1 μg of total RNA was reacted with a RevertAid reverse transcriptase (Thermo Fisher Scientific), using the following primers: PA347 (for *girAB* transcripts), PA346 (*girC*), PA704 (*girX*), and PA349 (*gerE*_*Bc*_). The obtained cDNA was amplified by 18 PCR cycles using the primer sets PA353/PA354 (for *girAB* cDNA), PA344/PA345 (for *girC*), PA 702/PA703 (for *girX*), and PA348/PA306 (for *gerE*_*Bc*_). PCR products were separated by agarose gel electrophoresis. Gel images were cropped, using Paintgraphic2 (SOURCENEXT).

### Preparation of DNA substrates for *in vitro* recombination

The DNA substrates for an *in vitro* recombination assay were generated by inserting the *attL*–tetracycline resistant gene (*tet*) –*attR* or *attB*–*tet*–*attG* constructs into the pMD20 T-vector (Takara Bio, Kyoto, Japan) to produce pMDTEx and pMDTIn, respectively. A DNA fragment containing *tet* was amplified from pHY300 PLK (Takara Bio) with the PA541/PA542 primer set and digested with *Eco*RI. A Taq polymerase reaction at 72 °C for 10 min without primers was performed to fill the over-hanging end produced by *Eco*RI and to add an adenine at the 3′-end. The resulting DNA fragment was ligated with the pMD20 T-vector using the TA-cloning method to produce a pMDT plasmid. DNA fragments containing *attL*, *attR*, *attB*, and *attG* were obtained by PCR using the primer sets PA770/PA771, PA772/PA773, PA770/PA773, and PA772/PA771, respectively. The *attR* and *attG* fragments were digested with *Eco*RI and *Bam*HI, and inserted into the *Eco*RI–*Bam*HI site of the pMDT, while the *attL* and *attB* fragments were digested with *Hin*dIII and *Nde*I and inserted into the *Hin*dIII–*Nde*I site of the pMDT to create the pMDTEx and pMDTIn plasmids, respectively. To construct pMDTIn-Bs, the *attB* region of pMDTIn (*attB*_*Bc*_) was replaced by *Hin*dIII/*Nde*I digestion following insertion of a DNA fragment containing the *gerE*-coding regions from *B. subtilis* 168 (*attB*_*Bs*_), which was amplified from the chromosomal DNA using the PA805/PA806 primer set. The plasmids were propagated in *E. coli* DH5α. The purified plasmids were used as substrates in the *in vitro* recombination assay.

### Preparation of GirC and GirX proteins

*girC* and *girX* were amplified by PCR with the primer sets PA705/PA706 and PA702/PA703, respectively. PCR products were digested with *Nde*I and *Xho*I, and cloned into the *Nde*I-*Xho*I site of the pET22b(+) vector (Merck Millipore, MW, USA) to obtain the pET-girC and pET-girX expression vectors. *E. coli* cells harboring the expression vectors were grown at 30 °C in LB medium containing 100 μg/ml ampicillin to an OD_600_ of 0.5. The recombinant GirC and GirX proteins with a his_6_ tag fused to the C-termini were induced by addition of 0.5 mM isopropyl β-D-1-thiogalactopyranoside (IPTG) at 30 °C for 3 hrs. The cells were then harvested by brief centrifugation and resuspended in a solution containing 50 mM sodium phosphate (pH 8.0), 0.3 M NaCl, 1× FastBreak reagent (Promega, WI, USA), 100 μg/ml lysozyme, and 100 μg/ml DNase I. To remove cell debris, the cell lysate was centrifuged at 20,400 g for 10 min and the supernatants were collected. The *E. coli* lysate containing his_6_-tagged GirC was loaded onto a HisTrap HP column (GE Healthcare), washed with 50 mM sodium phosphate (pH 7.4), 1 M NaCl, and 10 mM imidazole, and then bound proteins were eluted with 50 mM sodium phosphate (pH 7.4), 0.3 M NaCl, and 500 mM imidazole. The eluted fraction was directly loaded to a Heparin HP column (GE Healthcare), washed with 50 mM sodium phosphate (pH 7.4) and 0.5 M NaCl, and GirC was eluted with 1 M NaCl. The *E. coli* lysate containing the his_6_-tagged GirX was loaded onto a TALON column (Takara Bio), washed with a solution containing 50 mM sodium phosphate (pH 8.0) and 0.3 M NaCl, and eluted with 250 mM imidazole. Protein concentrations were measured using the Bradford quantification kit (BioRad, CA, USA) with BSA as the standard.

### *In vitro* recombination assay

Unless stated otherwise, the DNA substrates (200 ng), pMDTEx, and pMDTIn, were reacted with GirC-His_6_ (1 μM) and GirX-His_6_ (4 μM) at 37 °C for 60 min in 10 μl of the reaction solution containing 10 mM Tris-HCl (pH 7.5), 250 mM NaCl, 0.1 mM MgCl_2_, and 0.1 mM DTT. The recombination reaction was stopped by the addition of 0.1% SDS and heated at 60 °C for 3 min. The recombination products were separated by 1% agarose gel electrophoresis. Gel images were cropped, using Paintgraphic2 (SOURCENEXT).

## Electronic supplementary material

Supplementary Information
