# Supplementary material for: A novel non prophage(-like) gene-intervening element within gerE that is reconstituted during sporulation in Bacillus cereus ATCC10987
Source: Sci Rep. 2017 Sep 12;7:11426. doi: 10.1038/s41598-017-11796-8 (PMC5595907; doi:10.1038/s41598-017-11796-8)
Supplement: Supplementary file 1 — Supplementary Information [file 41598_2017_11796_MOESM1_ESM.pdf]

# Supplementary Information

## A novel non prophage(-like) gene-intervening element within *gerE* that is reconstituted during sporulation in *Bacillus cereus* ATCC10987.

**Kimihiro Abe[1], Shinya Shimizu[2], Shuhei Tsuda[2], and Tsutomu Sato[1,2*]**

[1]Research Center of Micro-Nano Technology, Hosei University, Koganei, Tokyo, Japan

[2]Department of Frontier Bioscience, Hosei University, Koganei, Tokyo, Japan

[*]To whom correspondence should be addressed. E-mail: t-sato@hosei.ac.jp

Department of Frontier Bioscience, Hosei University, Koganei 184-8584, Tokyo, Japan

Phone: (+81) 423877008; Fax: (+81) 423877002.

## Supplementary Methods

**Detection of prophage elements in *Bacillus cereus* ATCC10987.**

The *B. cereus* ATCC10987 genomic sequence (GenBank accession number, NC_003909.8) was scanned using an online tool, PHASTER (PHAge Search Tool Enhanced Release; http://phaster.ca/)[1,2], to detect prophage elements. Criteria for scoring the prophage regions are described on the web site and in the papers by Zhou *et al.* [1] and Amdt *et al.* [2].

**Gene complementation test for *gerE*.** To construct the *B. subtilis gerE* deletion mutant strain, GEd, two DNA fragments corresponding to the upstream and downstream franking regions of *gerE* were amplified from the *B. subtilis* 168 genome using the PA266/PA267 and PA268/PA269 primer sets. A DNA fragment containing the spectinomycin resistance gene (*spc*) was amplified from the pUCS191 plasmid vector[3], using the PA262/PA263 primer set. Three DNA fragments were combined and amplified by over-extension PCR with the PA266/PA269 primer set, and the resulting *spc* gene cassette was introduced into *B. subtilis* strain 168 through natural competence. Transformants obtained by double crossing-over homologous recombination at the *gerE* locus were selected on LB plates containing 100 µg/ml spectinomycin. To generate plasmids carrying the *B. cereus gerE* genes, DNA fragments containing the 5′-*gerE* and composite *gerE* ($gerE_{Bc}$) genes were obtained by PCR of chromosomal DNA from the vegetative and sporulating cells of *B. cereus* ATCC10987 using the primer sets PA271/PA272 and PA271/PA273, respectively. PCR fragments were digested by *Eco*RI and *Bam*HI and cloned into the *Eco*RI-*Bam*HI site of the integration vector, pMF20[4]. The resultant plasmids, pMF-5′gerE and pMF-$gerE_{Bc}$, were introduced into GEd to obtain the strains GEd-5 and GEd-C, respectively. Transformants were generated by a double-crossing over event at *amyE* and selected on LB plates containing 5 µg/ml chloramphenicol and 100 µg/ml spectinomycin.

The 168-Z, GEd-Z, GEd-5Z and GEd-CZ strains carrying a $P_{cotG}$–*lacZ* construct were constructed, using pMUTIN-T3[5]. A plasmid carrying the $P_{cotG}$–*lacZ* construct (pCG-Z) was generated by inserting the *B. subtilis cotG* promoter into. A DNA fragment containing the *B. subtilis cotG* promoter was amplified from the *B. subtilis* chromosomal DNA with the P278/P279 primer set, digested with *Hin*dIII and *Bam*HI, and cloned into the *Hin*dIII/*Bam*HI site of pMUTIN-T3. pCG-Z was introduced into *B. subtilis* 168, GEd, GEd-5, and GEd-C to create 168-Z, GEd-Z, GEd-5Z, and GEd-CZ, respectively.

**Strain construction of 168Gin.** pMF20 was linearized by inverse PCR with the PA365/PA366 primer set and gel-purified. A region from 5′-*gerE* to *gerE*-3′ encompassing the whole *gin* element sequence of *B. cereus* was amplified by PCR using the PA246/PA249 primer set. The PCR product was ligated with the linearized pMF20 using Gibson assembly (NEB), and directly introduced into *B. subtilis* 168.

**Strain construction of the 168Gin deletion series.** An erythromycin resistance gene, *erm*, was amplified from pUCE191[3] with the PA156/PA157 primer set. The upstream (left arm) and downstream (right arm) regions of *girAB* were obtained by PCR using the primer sets, PA458/PA459 and PA460/PA339, respectively. The *erm* cassette for the *girAB* deletion was generated using over-extension PCR with the PA458/PA339 primer set. A kanamycin resistance gene, *kan*, was amplified from pJM114[6] with the PA466/PA467 primer set. The upstream and downstream regions of *girC* were obtained by PCR using the primer sets, PA462/PA463 and PA464/PA465, respectively. The *kan* cassette for *girC* deletion was generated by over-extension PCR with the PA462/PA465 primer set. The *erm* and *kan* cassettes were introduced into 168Gin to obtain the *girAB*- and *girC*-deletion mutant (designated GABd and GCd, respectively). To delete the region from *girB* to *BCE4614* (GR1), the upstream and downstream regions were obtained by PCR with the primer sets, PA680/PA684 and PA460/PA339, respectively, and ligated with the *kan* gene fragment by over-extension PCR with the PA680/PA339 primer set. To delete the region from *BCE4615* to *BCE4625* (GR2), the upstream and downstream regions were obtained by PCR with the primer sets PA280/PA671 and PA685/PA681, respectively, and ligated with the *kan* gene fragment by over-extension PCR with the PA280/PA681 primer set. To delete the region from *girB to BCE4625* (GR3), the upstream and downstream regions were obtained by PCR with the primer sets, PA688/PA687 and PA460/PA339, respectively, and ligated with the *kan* gene fragment by over-extension PCR with the PA688/PA339 primer set. The kanamycin gene cassettes were introduced into 168Gin to obtain the GR1–3 strains, and the transformants were selected on the LB plates containing 5 μg/ml kanamycin and 5 μg/ml chloramphenicol. A DNA fragment containing the *BCE4620* gene (*girX*) was generated by PCR with the PA690/PA691 primer set, digested by *Bam*HI, and inserted into the *Bam*HI site within the pTCE1 integration vector[7]. The resulting plasmid, pTCE-girX, was introduced into GR2 to create GR2X via a double-crossing over recombination at the *thrC* locus, and the transformants were selected on the LB plates containing 0.3 μg/ml erythromycin, 5 μg/ml kanamycin, and 5 μg/ml chloramphenicol.

**Construction of GC-i.** A DNA fragment corresponding to 5′-portion of *girC* was amplified from the *B. cereus* ATCC10987 genome, using the PA623/PA345 primer set. PCR product was digested with *Hin*dIII and *Bam*HI and inserted into the *Hin*dIII–*Bam*HI site of pMUTIN-T3, The resulting vector, pGirC-IND, was introduced into 168Gin by natural competence to create a *girC*-inducible strain, GC-i. The transformants were selected on LB-agar plates containing 0.3 μg/ml erythromycin.

## Supplementary Tables

**Table S1.** Genes identified in the *gin* element of *B. cereus* ATCC 10987.

| Gene ID | Position[a] | Size (aa) | Possible function | Similar protein | | |
|---|---|---|---|---|---|---|
| | | | | Organism | Gene ID | Identity (%) |
| BCE_4594 | 4256674←4256802 | 42 | GerE (C-terminus) | *Bacillus cereus* FT9 | BcrFT9_03554 | 100 |
| BCE_4595 | 4256971→4257342 | 123 | unknown | *Bacillus cereus* VD166 | IK9_03861 | 68 |
| BCE_4596 | 4257370←4260009 | 879 | SbcC; exnuclease | *Bacillus cereus* VD166 | IK9_03862 | 74 |
| BCE_4597 | 4260040←4260639 | 199 | unknown | *Bacillus cereus* VD166 | IK9_03863 | 76 |
| BCE_4598 | 4260629←4262134 | 501 | unknown | *Bacillus cereus* VD166 | IK9_03864 | 73 |
| BCE_4599 | 4262494←4263957 | 487 | GirC; site-specific recombinase | *Bacillus cereus* Q1 | BCQ_4297 | 81 |
| BCE_4600 | 4264150←4264260 | 36 | unknown; unique | | | |
| BCE_4601 | 4264300←4265826 | 508 | unknown; unique | *Bacillus cereus* F528-94 | TU52_00505 | 72 |
| BCE_4602 | 4265826←4266884 | 352 | nucleoid-associated protein | *Bacillus cereus* F528-94 | TU52_00510 | 72 |
| BCE_4603 | 4266979←4267257 | 92 | unknown | *Bacillus cereus* F528-94 | TU52_00515 | 77 |
| BCE_4604 | 4267501←4268295 | 264 | type II restriction endonuclease | *Bacillus cytotoxicus* CVUAS 2833 | TU51_18135 | 95 |
| BCE_4605 | 4268401←4269489 | 362 | DNA-cytosine methyltransferase | *Bacillus cytotoxicus* CVUAS 2833 | TU51_18130 | 93 |
| BCE_4606 | 4270085←4270213 | 42 | unknown; unique | | | |
| BCE_4607 | 4270450←4272030 | 526 | GirB; site-specific recombinase | *Bacillus cereus* AH820 | BCAH820_4586 | 100 |
| BCE_4608 | 4272035←4273570 | 511 | GirA; site-specific recombinase | *Bacillus cereus* AH820 | BCAH820_4587 | 100 |
| BCE_4609 | 4273782→4274129 | 115 | transcriptional regulator | *Bacillus cereus* AH820 | BCAH820_4589 | 100 |
| BCE_4610 | 4274268→4275929 | 553 | RecJ; single-stranded-DNA-specific exonuclease | *Bacillus cereus* AH820 | BCAH820_4590 | 100 |
| BCE_4611 | 4276036←4276908 | 290 | DNA-binding protein | *Bacillus cereus* AH820 | BCAH820_4591 | 100 |
| BCE_4612 | 4276960←4277052 | 30 | unknown; unique | | | |
| BCE_4613 | 4277167→4277508 | 113 | transcriptional regulator | *Bacillus cereus* AH820 | BCAH820_4592 | 100 |
| BCE_4614 | 4277651→4278016 | 121 | unknown | *Bacillus cereus* AH820 | BCAH820_4593 | 100 |
| BCE_4615 | 4278186←4278287 | 33 | unknown | *Bacillus thuringiensis* MC28 | MC28_F077 | 61 |
| BCE_4616 | 4278441→4279550 | 369 | PemK-like toxin of type II toxin-antitoxin system | *Bacillus cereus* AH820 | BCAH820_4594 | 100 |
| BCE_4617 | 4279699←4279881 | 60 | unknown; unique | *Bacillus cereus* AH820 | BCAH820_4595 | 100 |
| BCE_4618 | 4280292←4281029 | 245 | unknown | *Bacillus cereus* AH820 | BCAH820_4596 | 100 |
| BCE_4619 | 4281098←4281475 | 125 | unknown | *Bacillus cereus* AH820 | BCAH820_4597 | 100 |
| BCE_4620 | 4281631←4281759 | 42 | GirX; recombination directionality factor for GirC | *Bacillus cereus* AH820 | BCAH820_4598 | 100 |
| BCE_4621 | 4281918←4282742 | 274 | unknown | *Bacillus cereus* AH820 | BCAH820_4599 | 100 |
| BCE_4622 | 4283117→4283500 | 127 | transcriptional regulator | *Bacillus cereus* AH820 | BCAH820_4600 | 100 |
| BCE_4623 | 4283518→4284285 | 255 | peptidase M78 | *Bacillus cereus* AH820 | BCAH820_4601 | 100 |
| BCE_4624 | 4284780←4285007 | 75 | unknown; unique | | | |
| BCE_4625 | 4285142←4285246 | 34 | unknown; unique | | | |
| BCE_4626 | 4285349←4285534 | 61 | GerE (N-terminus) | *Bacillus cereus* FT9 | BcrFT9_03563 | 100 |

[a] Arrows indicate the direction of transcription.

**Table S2.** Strains and plasmids.

| Strain or plasmid | Genotype and/or relevant features | Source, reference, or construction |
|---|---|---|
| **Strain** | | |
| *Bacillus cereus* | | |
| ATCC10987 | wild type strain | BGSC |
| | | |
| *Bacillus subtilis* | | |
| 168 | *trpC2* | [8] |
| GEd | *trpC2*, Δ*gerE::spc*; Spc$^r$ | This study |
| GEd-5 | GEd, *amyE*::pMF-5'gerE; Spc$^r$, Cm$^r$ | pMF-5'gerE→GEd |
| GEd-C | GEd, *amyE*::pMF-gerE$_{Bc}$; Spc$^r$, Cm$^r$ | pMF-gerE$_{Bc}$→GEd |
| 168-Z | *trpC2, cotG*::pMUTIN-T3, P$_{cotG}$–*lacZ*; Em$^r$ | pMUTCG→168 |
| GEd-Z | GEd, *cotG*::pMUTIN-T3, P$_{cotG}$–*lacZ*; Spc$^r$, Cm$^r$, Em$^r$ | 168-Z→GEd |
| GEd-5Z | GEd-5, *cotG*::pMUTIN-T3, P$_{cotG}$–*lacZ*; Spc$^r$, Cm$^r$, Em$^r$ | 168-Z→GEd-5 |
| GEd-CZ | GEd-C, *cotG*::pMUTIN-T3, P$_{cotG}$–*lacZ*; Spc$^r$, Cm$^r$, Em$^r$ | 168-Z→GEd-C |
| 168Gin | *trpC2*, *amyE*::5'-*gerE-gin-gerE*-3'; Cm$^r$ | This study |
| GABd | 168Gin, Δ*girAB*::*erm*; Cm$^r$, Em$^r$ | This study |
| GCd | 168Gin, Δ*girC*::*kan*; Cm$^r$, Km$^r$ | This study |
| GR1 | 168Gin, Δ(*girB*–*BCE4614)*::*kan*; Cm$^r$, Km$^r$ | This study |
| GR2 | 168Gin, Δ(*BCE4615*–*BCE4625*)::*kan*; Cm$^r$, Km$^r$ | This study |
| GR3 | 168Gin, Δ(*girB*–*BCE4619*)::*kan*; Cm$^r$, Km$^r$ | This study |
| GR2X | GR2, *thrC*::*girX*; Cm$^r$, Km$^r$, Em$^r$ | pTCE-girX→GR2 |
| GC-i | 168Gin, P$_{spac}$–*sprB*, *lacI$^q$*, *erm*; Cm$^r$, Em$^r$ | pGirC-IND→168Gin |
| BsINDB | *trpC2*, P$_{spac}$–*sprB, lacI$^q$*, *erm*; Em$^r$ | [9] |
| | | |
| *Escherichia coli* | | |
| DH5α | F$^-$, Φ80d*lacZ*ΔM15, Δ(*lacZYA-argF*)*U169*, *deoR*, *recA1*, *endA1*, *hsdR17*(r$_K^-$, m$_K^+$), *phoA*, *supE44*, *λ$^-$*, *thi-1*, *gyrA96*, *relA1* | Takara Bio |
| BL21 SHuffle T7 Express *lysY* | MiniF, *lysY* / *fhuA2*, *lacZ::T7 gene1*, *lon*, *ompT*, *ahpC*, *gal*, *att::pNEB3-r1-cDsbC* (*lacI$^q$*), Δ*trxB*, *sulA11*, *R*(*mcr-73::miniTn10*-Tet$^s$)*2*, *dcm*, *R*(*zgb-210::Tn10*-Tet$^s$), *endA1*, Δ*gor*, Δ*(mcrC-mrr)114::IS10*; Cm$^r$, Spc$^r$ | NEB |
| | | |
| **Plasmid** | | |
| pUCE191 | pUC19 carrying *erm* | [3] |
| pUCS191 | pUC19 carrying *spc* | [3] |
| pMF20 | *B. subtilis* integration vector (*amyE*) | [4] |
| pMF-5'gerE | pMF20 carrying 5'-*gerE* | This study |
| pMF-gerE$_{Bc}$ | pMF20 carrying *gerE$_{Bc}$* | This study |
| pMUTIN-T3 | P$_{spac}$–*lacZ*, *lacI$^q$*, *erm*, *bla* | [5] |
| pCG-Z | pMUTIN-T3 carrying a P$_{cotG}$–*lacZ* construct | This study |
| pGirC-IND | pMUTIN-T3 carrying a P$_{spac}$–*girC'* construct | This study |
| pJM114 | cloning vector carrying *kan* | [6] |
| pTCE1 | *B. subtilis* integration vector (*thrC*) | [7] |
| pTCE-girX | pTCE1 carrying *girX* | This study |
| pET22b(+) | expression vector, *bla* | Merck Millipore |
| pET-girC | pET22b(+) carring *girC* | This study |
| pET-girX | pET22b(+) carring *girX* | This study |
| pMD20 | Cloning vector (T-vector), *bla* | Takara Bio |
| pMDT | pMD20 carrying *tet* | This study |
| pMDTEx | pMD20 carrying the *attL*-*tet*-*attR* construct | This study |
| pMDTIn | pMD20 carrying the *attB-tet*-*attG* construct | This study |
| pMDTIn-Bs | pMD20 carrying the *gerE$_{Bs}$*-*tet*-*attG* construct | This study |

**Table S3.** Primers used in this study.

| Primer | Sequence (5′-3′)[a] | Location[b] |
|---|---|---|
| PA156 | ggcccaaacgcatggTAAACGTATATAGAT | pUCE191 *ermC* sense strand -370 |
| PA157 | cggccatctccttggTCGCGCGTTTCGGTG | pUCE191 *ermC* anti-sense strand +973 |
| PA246 | TTCATTCGTACAGCTAATTGGAATT | *Bce* 5'-*gerE* sense strand -376 |
| PA247 | CATACTTATCCAATACTTCATAAAA | *Bce* 5'-*gerE* anti-sense strand +462 |
| PA248 | CTCAATGTTTGGTTCTGTCCCTAC | *Bce gerE*-3' sense strand -277 |
| PA249 | CACGAGGTATCCCGTTCGGAATAGA | *Bce gerE*-3' anti-sense strand +405 |
| PA262 | ggcccaaacgcatggTCGCGCGTTTCGGTGATGAC | pUCS *spc* sense strand -348 |
| PA263 | cggccatctccttggGCGGTATTTCACACCGCATA | pUCS *spc* anti-sense strand +1072 |
| PA266 | ACAATGGCTAAACATGTAAGCCCGTATGAA | *Bsu gerE* sense strand -1471 |
| PA267 | ccatgcgtttgggccGTATTGTAACCCTCCTTGCT | *Bsu gerE* anti-sense strand -20 |
| PA268 | ccaaggagatggccgTCCTTGCCGGTATTCCTTCT | *Bsu gerE* sense strand +245 |
| PA269 | ACTGATGATCCGGGGCAATCGGAGACTGGT | *Bsu gerE* anti-sense strand +1596 |
| PA271 | gagtgaattcAAGGAGTGTCTGCACTGTATAAAAG | *Bce gerE* sense strand -133 |
| PA272 | ctcaggatccATTATGAAAATAAGTATCAAACACA | *Bce gerE* anti-sense strand +178 |
| PA273 | gttcggatccTTCATATAGAACAAGAAACAGACAG | *Bce gerE*-3' anti-sense strand +169 |
| PA278 | ttccaagcttGACATCGAAGAAGCGGTGAAATCCG | *Bsu cotG* sense strand +46 |
| PA279 | ttgtggatccGAGATTTTTTGTGAGAACAGAATGA | *Bsu cotG* anti-sense strand +193 |
| PA280 | GGCGTACTGCCTGAACGAGAAGCTATCACC | *Bsu amyE* anti-sense strand +996 |
| PA306 | AAAAGTTCACCTGCAATTTCCTTCG | *Bce* 5'-*gerE* anti-sense +86 |
| PA309 | TTTCATCACGACTTGCGATGAATCTTTCGA | *Bce gerE*-3' sense strand -671 |
| PA310 | ATCTAATACAAAGATTAAAAACTCCTCAAA | *Bce gerE*-3' anti-sense -70 |
| PA339 | ccaaagatctTCATTAAATACTTAAATATTCAGTT | *Bce BCE4604* anti-sense strand +774 |
| PA344 | atgtaagcttATCAACTGATCGTGATGAACAAATT | *Bce girC* sense strand +54 |
| PA345 | cgctggatccCACGTTTGTTCATCTACAACTTTCG | *Bce girC* anti-sense strand +788 |
| PA345 | cgctggatccCACGTTTGTTCATCTACAACTTTCG | *Bce girC* anti-sense strand +788 |
| PA346 | TGAATTGGTACATGGTTTATGCATCCCGATTTTCC | *Bce girC* anti-sense strand +1000 |
| PA347 | TAAAGTTACAGTTCTTGCAATCATATATACGTCTT | *Bce girB* anti-sense strand +1011 |
| PA348 | AGCAAGGAGGGTTATACCAGTTGAAGGAAAAAGCG | *Bce* 5'-*gerE* sense strand +15 |
| PA349 | TTATAACTCGAGCTCTCCCATGCGAAGAAG | *Bce gerE*-3' anti-sense strand +100 |
| PA353 | ataaaagcttTTGAGTATAGTATGAAAAAACGTAT | *Bce girA* sense strand +119 |
| PA354 | taaaggatccTTTCTAGTAAAAATTGAATTGTTTC | *Bce girA* anti-sense strand +853 |
| PA365 | ttagctgtacgaATGAATCAGCGCGGCTCACATGG | *Bsu amyE* sense strand +1232 |
| PA366 | acgggatacctcgtgTCCAGTTTGGAATACTCTTA | *Bsu amyE* anti-sense strand +465 |
| PA458 | TAAATATGAATAAATGTGAGTTTGTTTGACA | *Bce girA* sense strand -570 |
| PA459 | ccatgcgtttgggccAGGTCGTTGCATCAATGTTT | *Bce girA* anti-sense strand +167 |
| PA460 | ccaaggagatggccgTAAATAATTCTTTAATTGAA | *Bce girB* sense strand +1575 |
| PA462 | TTGAAATTACTCGTAGAAAATGTACTTC | *Bce girC* sense strand -1475 |
| PA463 | ccatgcgtttgggccAAATCAATCACCCACTTCTG | *Bce girC* anti-sense strand -20 |
| PA464 | ccaaggagatggccgAGACTTGTTTTATTGGTTAT | *Bce girC* sense strand +1484 |
| PA465 | ATAGTAATACATGATTTTAGAGAGCTTGTT | *Bce girC* anti-sense strand +2871 |

*Continued on the next page.*

| | | |
|---|---|---|
| PA466 | ggcccaaacgcatggGATATCGCCGTATGTAAGGA | pJM114 *kan* -316 |
| PA467 | cggccatctccttggTAGACATCTAAATCTAGGTA | pJM114 *kan* +796 |
| PA541 | ggcccaaacgcatggATATTGTTGTATAAGTGATG | pHY300 *tet* sense strand -172 |
| PA542 | cggccatctccttggCCGGGAATTCCTGTTATAAA | pHY300 *tet* anti-sense strand +1425 |
| PA623 | ggccaagcttAAGTAAGGAGACTTTGCTGTCATTT | *Bce girC* sense strand -292 |
| PA671 | ccatgcgtttgggccTCTTTTATTCCATTATAATA | *Bce* 5'-*gerE* anti-sense +256 |
| PA680 | TGTAATGTCTAGGTATAACAAACTCGTTGAA | *Bce BCE4614* anti-sense strand +2326 |
| PA681 | AATCATTTATTTGATGATTTGATGGCACTG | *Bce BCE4614* sense strand -1971 |
| PA684 | ccatgcgtttgggccCATATCTATATGGCTATGAT | *Bce BCE4614* sense strand +460 |
| PA685 | ccaaggagatggccgATCATAGCCATATAGATATG | *Bce BCE4614* anti-sense strand +441 |
| PA687 | ccatgcgtttgggccACATTGAAATTCACTTTTCA | *Bce BCE girX* anti-sense strand +286 |
| PA688 | TGAATTTTTATAATATATTGTTATTGTTTAGCCAT | *Bce BCE4621* sense strand -240 |
| PA690 | aggtaggatccATATAGAGTTATTTCCTATAAATT | *Bce girX* sense strand -124 |
| PA691 | cataggatccATATGACGGCTTAACTACCTTTGAT | *Bce* girX anti-sense strand +228 |
| PA702 | ggagatatacatatgACTAAAAAATCATCCCCACT | *Bce girX* sense strand +23 |
| PA703 | gtggtggtgctcgagAACAGGAGATTTCTCTTCTT | *Bce girX* anti-sense strand +107 |
| PA704 | AAAATAAAATCCCCTGATTAAACAG | *Bce girX* anti-sense strand +122 |
| PA705 | ggagatatacatatgGAATCGTATGCAGTTTATGT | *Bce girC* sense strand +23 |
| PA706 | gtggtggtgctcgagCGGTTTCTCAAATGTATATG | *Bce girC* anti-sense strand +1442 |
| PA770 | actaagcttGAGAGAGAGAGAAGTATTTG | *Bce* 5'-*gerE* sence strand +61 |
| PA771 | aaacatatggaattcTGAAATCTTCGTTCCATTTT | *Bce* 5'-*gerE* anti-sence strand +222 |
| PA772 | tttggatccATCTTTGTATTAGATACAAT | *Bce gerE*-3' sence strand -36 |
| PA773 | ccgcatatggaattcAAAGTCGGCTTTCTTTTTAT | *Bce gerE*-3' anti-sence strand +126 |
| PA805 | actaagcttTTGAAGGAGAAAGAATTTCAATCG | *Bsu gerE* sense strand +24 |
| PA806 | ccgcatatggaattcTTAAAGCTCTAGCTCACCCA | *Bsu gerE* anti-sense strand +205 |

[a] Inserted and/or mutated nucleotides are written in lower case.

[b] Location numbers indicate the distance from the 3′-end nucleotide of the primer to the first nucleotide of the gene. *Bsu* and *Bce* indicate *B. subtilis* 168 genes and *B. cereus* ATCC10987 genes, respectively.

## Supplementary Figures

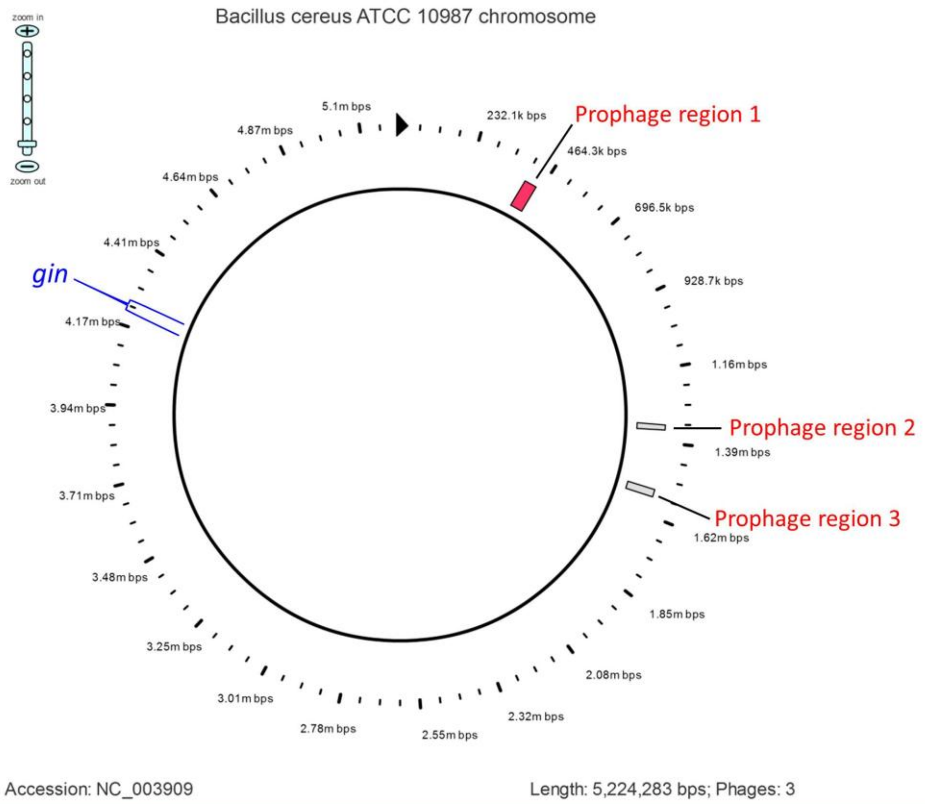


**Total:** 3 prophage regions have been identified, of which 1 regions are intact, 2 regions are incomplete, and 0 regions are questionable.

| Region | Region Length | Completeness | Score | # Total Proteins | Region Position | Most Common Phage | GC % | Details |
|---|---|---|---|---|---|---|---|---|
| 1 | 43.9Kb | intact | 130 | 23 | 403390-447381 | PHAGE_Bacill_Fah | 40.58% | Show |
| 2 | 20.2Kb | incomplete | 40 | 24 | 1333290-1353520 | PHAGE_Escher_phAPEC8 | 36.23% | Show |
| 3 | 25.7Kb | incomplete | 20 | 8 | 1541412-1567138 | PHAGE_Ectoca_1 | 35.30% | Show |

Intact (score > 90)
Questionable (score 70-90)
Incomplete (score < 70)

| | |
|---|---|
| **Region:** | The number assigned to the region. |
| **Region Length:** | The length of the sequence of that region (in bp). |
| **Completeness:** | A prediction of whether the region contains a intact or incomplete prophage based on the above criteria. |
| **Score:** | The score of the region based on the above criteria. |
| **# Total Proteins:** | The number of ORFs present in the region. |
| **Region Position:** | The start and end positions of the region on the bacterial chromosome. |
| **Most Common Phage:** | The phage(s) with the highest number of proteins most similar to those in the region. |
| **GC %:** | The percentage of GC nucleotides of the region. |

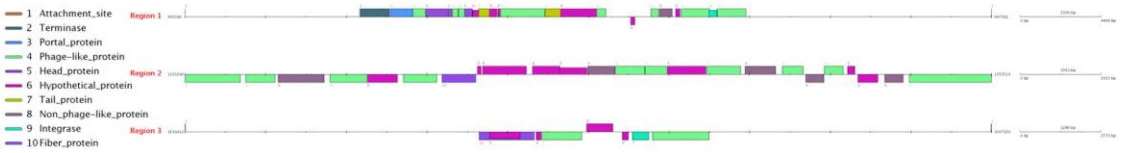


**Figure S1.** Prophage regions and the *gin* element in the *Bacillus cereus* ATCC10987 genome. A prophage search program, PHASTER (http://phaster.ca/), detected three regions in the *B. cereus* genome as prophages but not the *gin* region due to the lack of phage-related genes (e.g. phage head, tail, and fiber genes).

```
GirA      1 ----MVMLKMKKTIAYYRSSTDLQE---NSIEMQQIKAFEYSMKKRITIDEEYVDKDVSARKKTLMQRPGMNKLMKEIEAGVVGSIIVYKRDRFARKLQE 93
GirB      1 MRLEDILQPGKRVAFYARYSTKD-----QGYEMQKHSVEVLLERYGCEIKKEYVDAGVSATKVPFEERPHLQKLICDAAEKKFDCIVVYKNDRIARHIEE 95
GirC      1 ---------MESYAVYVRVSTDR-DEQISSVENQIDICRNWLERNGFKWDERCIYKDEGISGTLFTDRPAIQLLLQKAKAKKINTVVFKSISRLARDLKD 90
SpoIVCA   1 -----------MIAIYVRVSTEEQAIKGSSIDSQIEACIKKAGTK--DVLKY---ADEGFSGEL-LERPALNRLREDASKGLISQVICYDPDRLSRKLMN 83
A118      1 ----------MKAAIYIRVSTQEQVEN-YSIQAQTEKLTALCRSK--DWDVYDIFIDGGYSGSN-MNRPALNEMLSKLHE--IDAVVVYRLDRLSRSQRD 84

GirA     94 HMQLYRLFQEYDIDVYFSSDSEVQMNYSPIGEYIEAILGSICENEGSLIAQRIQETKIAKFLSGKYAG---NLPYGYEKGEPNADG----QVNILQVSHE 186
GirB     96 HDKFRKQMCEMNMHVVVSSSHELYTVG---EIVPQTVKDGLTRIEAALIQERTKDTFHSKAKRGAWLGGKAAYGYEYRVIEENNGGIKNRIEQFVDVEQE 192
GirC     91 -SLEIREVFLGHGVRIISVEEGYDSTKAGKNDMAFELWSLFSAQYSRTLSSSITAALAVKVRRGEHIG---KVPYGYNRVNQ----------KLVINKEE 176
SpoIVCA  84 -QLIIDDELRKRNIPLIFVNGEYANS--PEGQLFFAMRGAISEFEKAKIKERTSSGRLQKMKKGMIIKDSKLYGYKFVKEKR----------TLEILEEE 170
A118     85 TITLIEEYFLKNNVEFVSLSETLDTSS-PFGRAMIGILSVFAQLERETIRDRMVMGKIKRIEAGLPLTTAKGRTFGYDVIDT----------KLYINEEE 173

GirA    187 IEVVKEIYNALLNKECKS---IQELCNDLNEKYKTEETK--DWDQSKVVNIVKNPIYHGLRVMNFGSKE-------------------YKKAYKNIKVV 261
GirB    193 QQVVKEIYRLFELGFG-----FQQIADEIKNKKWDERIK---WTKERIRYIITNPFYSGLITMNRYYKGVLNNKS-----------EWIKGKVPNMNPIF 273
GirC    177 AEVVQRMYKWYNSGWG-----YKKITNELNKLGVRSKTN-KVWQMTSVQRILQNPIYKGTFILNQYTNVKIGGKK----KQIRNPKEKWFIFTNHHPKVV 266
SpoIVCA 171 AKIIRMIFNYFTDHKSPFFGRVNGIALHLTQMGVKTKKGAKVWHRQVVRQILMNSSYKGEHRQYKYDTEGSYVSKQAGNKSIIKIRPEEEQITVTIPAIV 270
A118    174 AKQLQLIYDIFEEEQS-----ITFLQKRLKKLGFKVRT-----------------YNRYNNWLTNDLYCGYVS-----------YKDKVHVKGIHEPII 239

GirA    262 EKTDWDIANEQLDKMIVKRETGKETIQ-FLLENILYCHECQKPLIAKKR---------MRSYQDYYIYECKEHDVCVEAGYIEVEILKHAKTFFHRLLDE 351
GirB    274 SQEYWEYIIGLFEERKERKIHSKDYSTPFLLRNILYCEHCKEAMTTKNQEPGGTKSKNKKEKKIRRIYDCKNCNFKLQAEMVHEQFRRKYLPYVLEQVTQ 373
GirC    267 DEQTWDQANQKNITLQKTKITPWNEFR--ELAKCAVCGSNMIIVQSHLK-----KKNGE--RTEWKYLKCSQYRRAGK-SGCINHVPIQYR--YFRQFII 354
SpoIVCA 271 PAEQWDYAQELLGQSKRKHLSISPHNY--LLSGLVRCGKCGNTMTGKKR-----KSHGK----DYYVYTCRKNYSGAKDRGCGKEMSENKLNRHVWGEIF 359
A118    240 SEEQFYRVQEIFTRMGKNPNMNRDSAS--LLNNLVVCSKCGLGFVHRRK-----DTMSRGKKYHYRYYSCKTYKHTHELEKCGNKIWR-------ADKLE 325

GirA    352 NFNELYERSTYQNVRKIKKLIGSQEMIIKGVTKKVGKLIDQWLNCQKMQREHE-----GNDCAKCQSNQHIGEIKEANEILKSEKRKLQHLDMRLYEIKN 446
GirB    374 GKKDQVELSIEKRLKKELSDLRSANRKLETEAIELNQQIEELNREIKLHYEQEIIQEEVLEFMKILMSHRSNKMHELNVIKDNQDELKKRMNRIEQTIKE 473
GirC    355 ELLIKKGESVTLKLQNNVEQGKKEKIKKLQQLMNVNEQKKQSLLDLYLEG----------LINKEEFEKKRNDLEKVVMKASQELFIIQQNDAVQIDIKT 444
SpoIVCA 360 KFITNPQKYVSFKEAEQSNH-LSDELELIEKEIEKTKKGRKRLLTLISLSDDDDLDIDEIKAQIIELQKKQNQLTEKCNEIQSKMKVLDDTSSSENALKR 458
A118    326 ELIIN--RVNNYSFASRNVD-KEDELDSLNEKLKIEHAKKKRLFDLYING----------SYEVSELDSMMNDIDAQINYYESQIEANEELKKNK-KIQE 411

GirA    447 FK-EKAKEWITHLECPKDILLLEKEERKRFYRDMIHCIQVDSWEYHIVFKHPFMLIQEVHQNSETV                                 511
GirB    474 KNYLPSHDLLSEFAQEN----VNPQKLRAFLLQFFKCVYITSNGELLMTVNNSLIES---------                                 526
GirC    445 I-----KEAFEQLHKRK----QELFH---VFNTLIEKIIIHQDGTIDITYTFEKP-----------                                 487
SpoIVCA 459 A-----IDYFQSIGADN----LTLEDKKTIVNFIVKEVTIVDSDTIYIETY---------------                                 500
A118    412 N-----LADLATVDFDS----LEFREKQLYLKSLINKIYIDG-EQVTIEWL---------------                                 452
```

**Figure S2.** Alignment of GirA, GirB, and GirC. Amino acid sequences of GirA, GirB, and GirC from *B. cereus* ATCC10987 are aligned with serine-type recombinases from Gram-positive bacteria: SpoIVCA from *B. subtilis* 168 and A118 Int from *Listeria innocua* Clip11262.

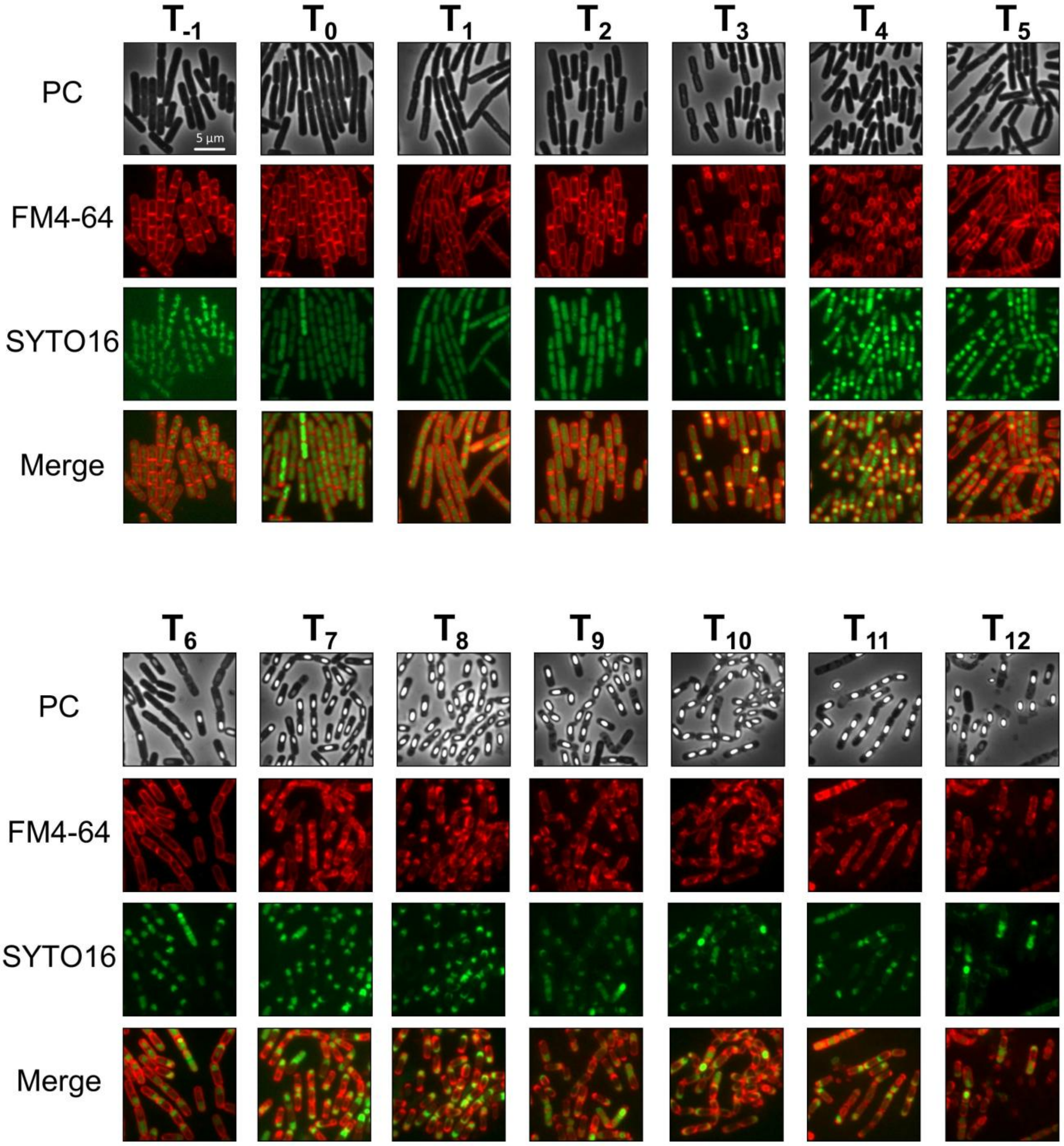


**Figure S3.** Morphological changes during *B. cereus* sporulation. *B. cereus* ATCC10987 cells were induced to sporulate by cultivation at 37°C in DSM. $T_0$ indicates the onset of the sporulation. PC, phase contrast; FM4-64, membrane staining with FM4-64; SYTO16, chromosomal DNA staining with SYTO16; Merge, merged images of FM4-64 and SYTO16.

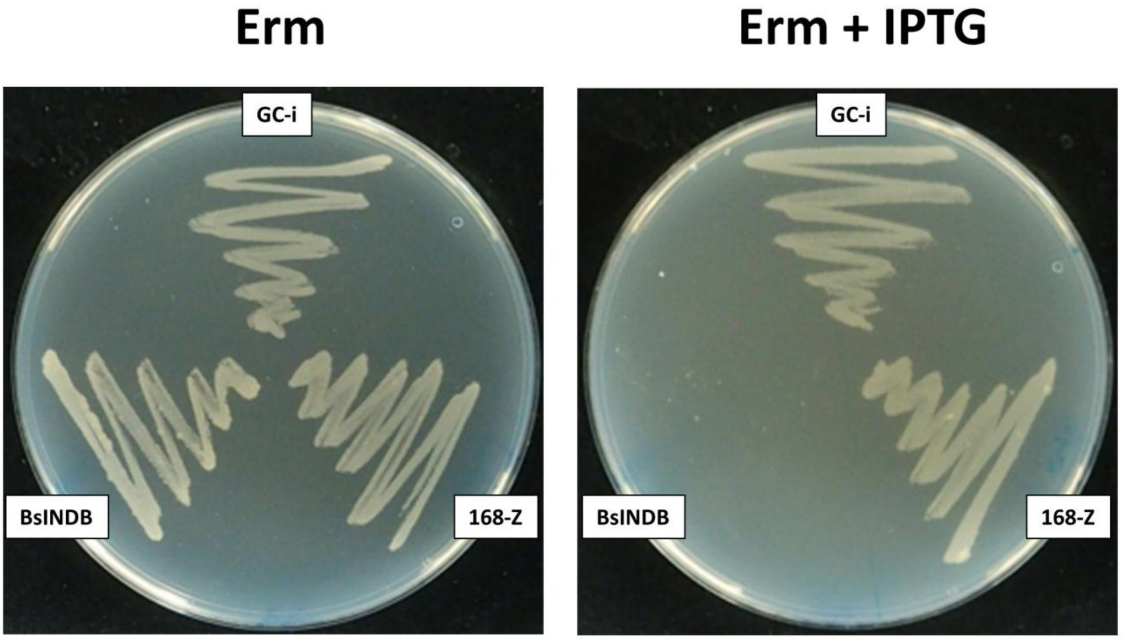


**Figure S4.** Examination of the induction of *gin* excision. A *girC*-inducible *B. subtilis* strain, GC-i, was constructed by the introduction of pMUTIN-T3 upstream of *girC* in 168Gin. GC-i induces *girC* excision following the addition of IPTG into the medium and is resistant to erythromycin because pMUTIN-T3 contains the *erm* gene; however, if *gin* was excised from the genome, it was expected to become sensitive to erythromycin. GC-i, 168-Z, and BsINDB were streaked onto LB plates containing erythromycin (0.5 µg/ml) in the absence or the presence of IPTG (1 mM), and cultured at 37°C overnight. 168-Z was used as a negative control in this experiment as it is resistant to erythromycin regardless of the presence of IPTG. BsINDB can induce SPß prophage excision following the addition of IPTG due to the *erm* gene in the prophage region[9]. The addition of IPTG results in BsINDB losing erythromycin resistance, while GC-i was shown to retain resistance.

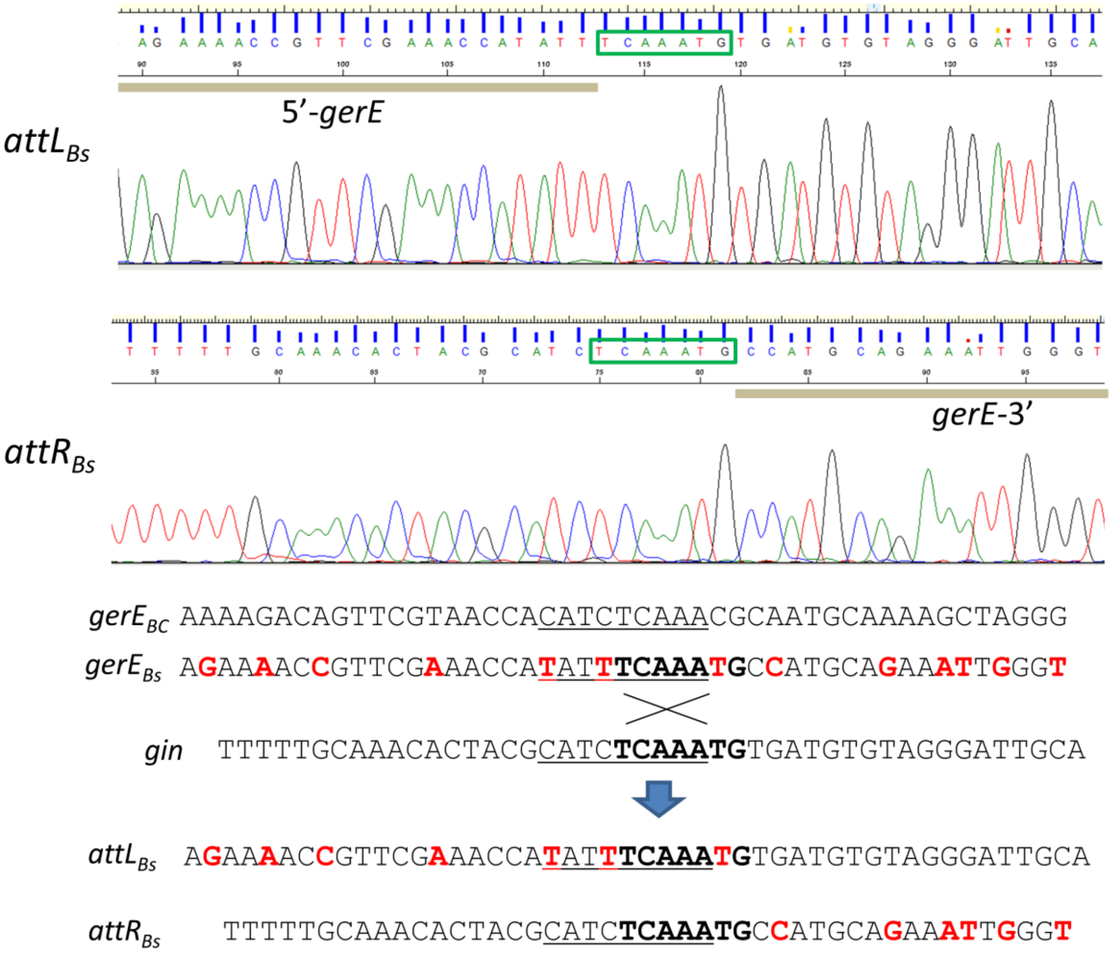


**Figure S5.** DNA sequences of *B. subtilis gerE* (*gerE*$_{Bs}$) junction sites. The integrative recombination products, *attL*$_{Bs}$ and *attR*$_{Bs}$, of the *in vitro* recombination between *gerE*$_{Bs}$ and *gin* were analyzed by DNA sequencing. Differences in the nucleotides between *gerE*$_{Bc}$ and *gerE*$_{Bs}$ are shown in red font. The consensus sequences between *gerE*$_{Bc}$–*gin* and between *gerE*$_{Bs}$–*gin* are underlined and in bold font, respectively.

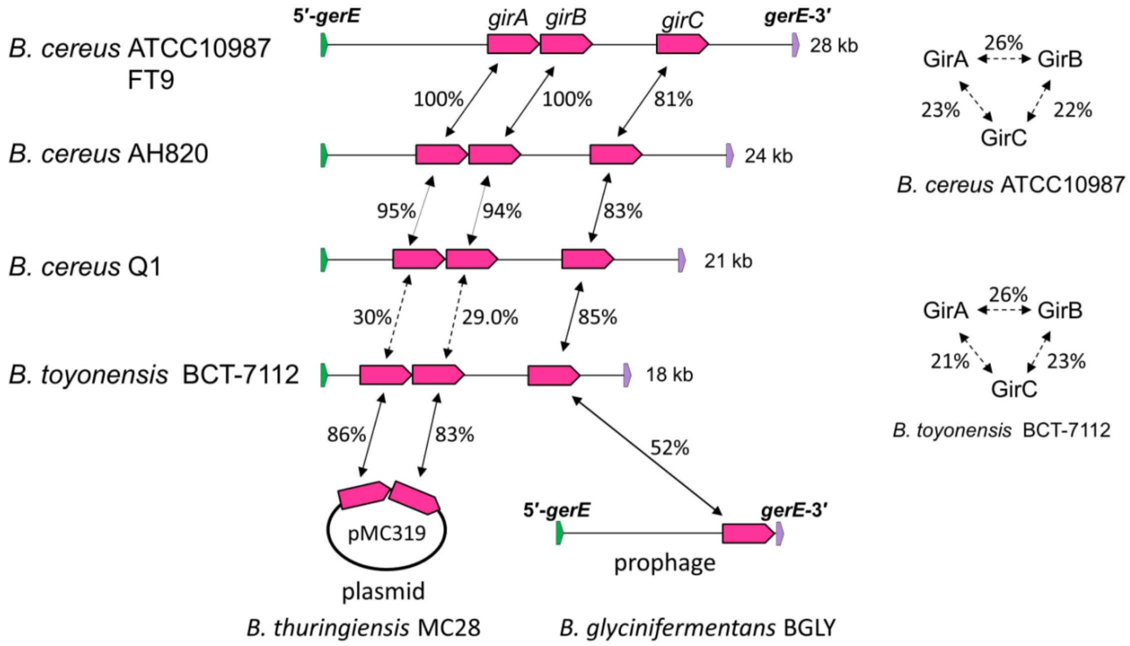


**Figure S6.** Conservation of GirA, GirB, and GirC in *B. cereus* and *B. toyonensis* strains. Schematic shows the gene organization of *gin* elements in *B. cereus* ATCC10987, AH820, and Q1 and *B. toyonensis* BCT-7112 strains. Scores indicate the % identity between the amino acid sequences of the recombinases.

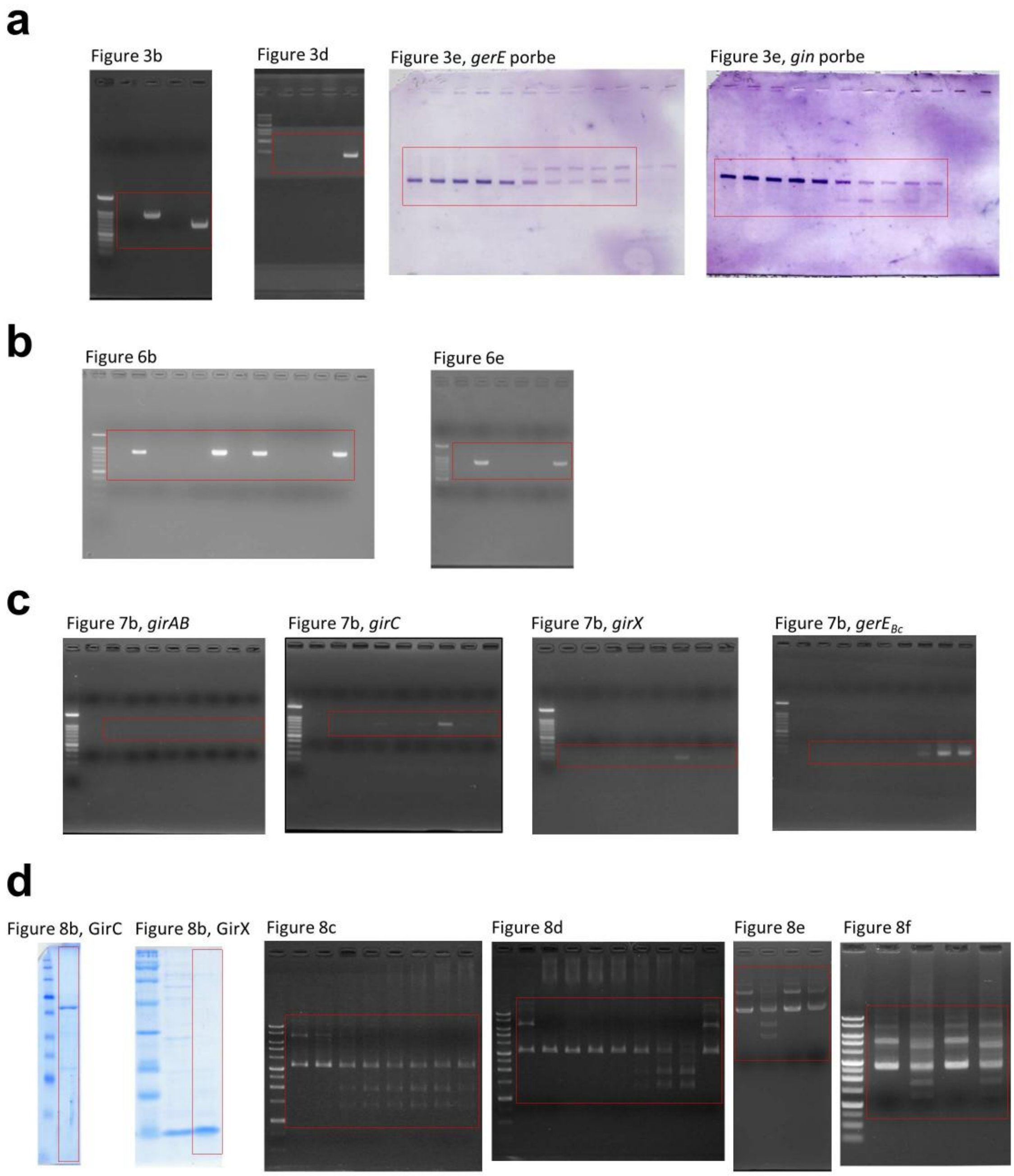


**Figure S7.** Full-sized gel and blot images. Raw gel and blot images of the main Figures 3, 6, 7, and 8 are shown in **a**, **b**, **c**, and **d**, respectively. The full-sized images were cropped as indicated by the red boxes. Southern blot and CBB-stained gel images were cropped and converted to gray scale. Processing of the images was performed, using Paintgraphic2 (SOURCENEXT, Tokyo, Japan).

## Supplementary References


[1] Zhou, Y., Liang, Y., Lynch, K.H, Dennis, J.J. & Wishart, D.S. PHAST: a fast phage search tool. *Nucleic Acids Res*. **39**, W347–352, doi:10.1093/nar/gkr485 (2011)

[2] Amdt, D. *et al.* PHASTER: a better, faster version of the PHAST phage search tool. *Nucleic Acids Res*. **44**, W16–21, doi:10.1093/nar/gkw387 (2016)

[3] Hosoya, S., Asai, K., Ogasawara, N., Takeuchi, M. & Sato, T. Mutation in *yaaT* leads to significant inhibition of phosphorelay during sporulation in *Bacillus subtilis*. *J. Bacteriol.* **184**, 5545–5553, doi:10.1128/JB.184.20.5545-5553.2002 (2002).

[4] Murakami, T., Haga, K., Takeuchi, M. & Sato, T. Analysis of the *Bacillus subtilis spoIIIJ* gene and its Paralogue gene, *yqjG*. *J. Bacteriol.* **184**, 1998–2004, doi:10.1128/JB.184.7.1998-2004.2002 (2002).

[5] Vagner, V., Dervyn, E. & Ehrlich, S. D. A vector for systematic gene inactivation in *Bacillus subtilis*. *Microbiology* **144 ( Pt 11)**, 3097–3104, doi:10.1099/00221287-144-11-3097 (1998).

[6] Marta, P. Integrational vectors for genetic manipulation in *Bacillus subtilis*. *In Bacillus subtilis and other gram-positive bacteria: biochemistry, physiology, and molecular genetics* (ed. A. L. Sonenshein, J. A. Hoch, and R. Losick), 615–624 (American Society for Microbiology), doi:10.1128/9781555818388.ch42 (1993).

[7] Imamura, D. *et al. spoIVH* (*ykvV*), a requisite cortex formation gene, is expressed in both sporulating compartments of *Bacillus subtilis*. *J. Bacteriol.* **186**, 5450–5459, doi:10.1128/JB.186.16.5450-5459.2004 (2004).

[8] Burkholder, P. R. & Giles, N. H., Jr. Induced biochemical mutations in *Bacillus subtilis*. *Am. J. Bot.* **34**, 345–348 (1947).

[9] Abe, K. *et al.* Developmentally-regulated excision of the SPβ prophage reconstitutes a gene required for spore envelope maturation in *Bacillus subtilis*. *PLoS Genet.* **10**, e1004636, doi:10.1371/journal.pgen.1004636 (2014).
